# Independent Pathways Can Transduce the Life-Cycle Differentiation Signal in *Trypanosoma brucei*

**DOI:** 10.1371/journal.ppat.1003689

**Published:** 2013-10-17

**Authors:** Balazs Szöőr, Naomi A. Dyer, Irene Ruberto, Alvaro Acosta-Serrano, Keith R. Matthews

**Affiliations:** 1 Centre for Immunity, Infection and Evolution, Institute for Immunology and Infection Research, School of Biological Sciences, University of Edinburgh, Edinburgh, United Kingdom; 2 Parasitology Department, Liverpool School of Tropical Medicine, Pembroke Place, Liverpool, United Kingdom; 3 Vector Biology Department, Liverpool School of Tropical Medicine, Pembroke Place, Liverpool, United Kingdom; University of California, Los Angeles, United States of America

## Abstract

African trypanosomes cause disease in humans and livestock, generating significant health and welfare problems throughout sub-Saharan Africa. When ingested in a tsetse fly bloodmeal, trypanosomes must detect their new environment and initiate the developmental responses that ensure transmission. The best-established environmental signal is citrate/*cis* aconitate (CCA), this being transmitted through a protein phosphorylation cascade involving two phosphatases: one that inhibits differentiation (*Tb*PTP1) and one that activates differentiation (*Tb*PIP39). Other cues have been also proposed (mild acid, trypsin exposure, glucose depletion) but their physiological relevance and relationship to *Tb*PTP1/*Tb*PIP39 signalling is unknown. Here we demonstrate that mild acid and CCA operate through *Tb*PIP39 phosphorylation, whereas trypsin attack of the parasite surface uses an alternative pathway that is dispensable in tsetse flies. Surprisingly, glucose depletion is not an important signal. Mechanistic analysis through biophysical methods suggests that citrate promotes differentiation by causing *Tb*PTP1 and *Tb*PIP39 to interact.

## Author Summary

African trypanosomes are important pathogens transmitted by tsetse flies in sub-Saharan Africa. Upon transmission, trypanosomes detect citrate and *cis*-aconitate in the bloodmeal, this inactivating a negative regulator of differentiation, the tyrosine phosphatase *Tb*PTP1. One *Tb*PTP1 substrate is another phosphatase, *Tb*PIP39, which is more active when phosphorylated (after *Tb*PTP1 inhibition) and promotes differentiation. These differentiation regulators have provided tools to monitor whether one or more environmental signals are used to initiate trypanosome development and their relevance *in vivo*. This is important because different studies over the last 30 years have disputed the physiological importance of different signals. Here we have, firstly, compared the efficacy of the different reported differentiation signals, establishing their relative importance. We then monitored *Tb*PIP39 phosphorylation to show that two signalling pathways operate: one signalled by citrate or mild acid, the other stimulated by external protease activity. Thereafter, we showed that, of these different signals, protease activity is dispensable for differentiation in tsetse flies. Finally, we used biophysical methods to investigate how citrate causes *Tb*PIP39 and *Tb*PTP1 to interact, enabling their regulatory cross-talk. These studies have established the importance of different developmental signals in trypanosomes, providing molecular insight into how the development signal is transduced within the pathogen.

## Introduction

Eukaryotic developmental events are a response to single or multiple external cues. Commonly, the existence of multiple cues ensures that cells do not embark prematurely on a developmental process that could damage their viability or fitness [1]. Additionally, the presence of multiple cues can lower the threshold at which cells respond to differentiation signals or refine their response, with inputs from distinct signalling pathways co-operating to generate a specific developmental outcome (e.g. [2], [3]). In this way, quite sophisticated perception mechanisms can contribute to ensure an appropriate and timely developmental response when cells encounter conditions where differentiation is the optimal survival response to a changing environment.

Although cell type differentiation is most studied in the programmed specialisation of metazoan cells as they form tissues or adapt for particular functions in the body, unicellular organisms can also undergo development in response to external signals. Exemplary of this are the differentiation responses of vector-borne parasites. These undergo development within distinct environments in their mammalian host, as well as during colonisation of their arthropod vectors to ensure their transmission [4], [5]. Among the best studied of these are the kinetoplastid parasites, representing the earliest branching extant eukaryotes [6] that are responsible for a range of tropical diseases such as visceral and cutaneous Leishmaniases (caused by different *Leishmania* spp.), American trypanosomiasis (‘Chagas' disease’, caused by *Trypanosoma cruzi*) and Human, and Animal, African trypanosomiasis (HAT, AAT, caused by *Trypanosoma brucei* [7]). For both *Leishmania* and *T. cruzi* several signals have been discovered that can trigger life-cycle differentiation including low temperature, pH balance [8] and, most recently in *Leishmania*, iron availability [9]. For African trypanosomes, citrate-*cis* aconitate (CCA) is routinely used in differentiation studies [10]. In *T. brucei* the optimal response to CCA requires the generation of a transmissible life cycle stage in the blood called ‘stumpy’ forms [11], which are non-dividing and show partial adaptation for conditions in the midgut of tsetse flies, the parasite's vector. For example, mitochondrial activity is elevated in the insect forms of the parasite compared to the proliferative bloodstream ‘slender’ forms, which are able to meet their energy demands through their metabolism of blood glucose through glycolysis alone [12]. Stumpy forms are sensitive to CCA through their expression of members of a surface carboxylate transporter family, called PAD (*P*roteins *A*ssociated with *D*ifferentiation) proteins [13]. These proteins transport CCA and at least one family member, PAD2, is elevated by the temperature reduction associated with passage from a homoeothermic to poikilothermic environment, sensitising parasites to physiological concentrations of CCA [14], [15]. The molecular details of the development from slender to stumpy forms are not well understood, although laboratory adapted lines that have lost the capacity to become stumpy (‘monomorphic lines’) can generate stumpy-like forms upon exposure to cell permeable cAMP and AMP analogues [16]–[18], or through *Tb*TORC4 depletion [19].

A key molecular regulator of trypanosome differentiation to tsetse midgut procyclic forms is the tyrosine phosphatase *Tb*PTP1 [20]. This enzyme acts as an inhibitor of parasite differentiation such that inactivation of the enzyme, or reduction of its levels by RNAi, elicits spontaneous differentiation in the absence of any trigger. Recently, a substrate of *Tb*PTP1 was identified as a serine/threonine *DxDxT* class phosphatase, *Tb*PIP39 [21]. This molecule is dephosphorylated on tyrosine 278 by *Tb*PTP1 and thereby held inactive, this inhibition being reinforced because *Tb*PIP39 itself promotes the activity of *Tb*PTP1. In the presence of CCA, however, the activation of *Tb*PTP1 by *Tb*PIP39 is reduced, such that *Tb*PIP39 becomes phosphorylated and differentiation is stimulated [21], [22]. This appears to be mediated *via* trafficking to the glycosome, a peroxisome-like organelle in trypanosomes that is the site of glycolysis and several other metabolic activities [23].

As in other kinetoplastid parasites, a number of other stimuli of trypanosome differentiation have been reported in addition to CCA, although the use of different cell lines and developmental forms has complicated interpretation of their efficacy or physiological relevance. These reported stimuli include (i) mild acid conditions [24], (ii) exposure of the parasite surface to limited protease digestion [25]–[27] and (iii) a reduction of glucose mediated through either the use of glucose depleted media [28], or exposure to phloretin, an inhibitor of glucose uptake [29]. Of these, both mild acid and protease treatment induce adenylate cyclase activity [30] and are restricted to transmissible stumpy forms, since bloodstream slender cells are not viable after exposure to these conditions [31], whereas glucose depletion has only been evaluated in monomorphic slender forms.

Here we have systematically investigated the importance of distinct stimuli of trypanosome differentiation, their physiological relevance and, in the case of CCA, mechanistic basis. Firstly, we used the phosphorylation of *Tb*PIP39 as a marker for operation of the citrate-dependent signalling cascade to determine whether a single, or multiple transduction pathways can elicit differentiation. This revealed that independent pathways for the initiation of trypanosome differentiation exist, one (stimulated by CCA and mild acid) operating *via* the *Tb*PTP1-*Tb*PIP39 signalling pathway, whereas an alternative stimulus, protease treatment, signals *via* an alternative route. Analysis of the contribution of each pathway in tsetse flies supports a physiological role for *Tb*PTP1-*Tb*PIP39 signalling, with insect trypsin activity not being required to stimulate parasite differentiation signal *in vivo*. Thereafter, we investigated the interaction between *Tb*PTP1 and *Tb*PIP39 and found that this is dependent upon the presence of the citrate differentiation signal, revealing a hitherto unexpected interplay between these molecules.

## Results

### Analysis of the efficacy of distinct differentiation stimuli

To date, a number of stimuli have been reported to induce trypanosome life cycle differentiation, but these have been analysed in different cell lines and under varying experimental conditions. Our identification of *Tb*PIP39 as a downstream substrate of the CCA-responsive regulator *Tb*PTP1 [21] provided tools to systematically investigate the pathways through which the development of trypanosomes is signalled. Therefore, we carefully analysed, in parallel, trypanosome differentiation stimulated *via* four reported triggers: *cis*-aconitate (CA), mild acid, pronase treatment or glucose deprivation using phloretin, a glucose transport inhibitor. Initially the efficacy of each pathway was evaluated such that bloodstream stumpy forms were exposed to 6 mM *cis*-aconitate, were incubated for 2 hours at pH 5.5, were treated with 4 units pronase from *Streptomyces griseus* for 10 minutes or were incubated in the presence of 100 µM phloretin. In each case, differentiation was monitored by flow cytometry for the expression of the developmental surface marker EP procyclin.

**Figure 1:**
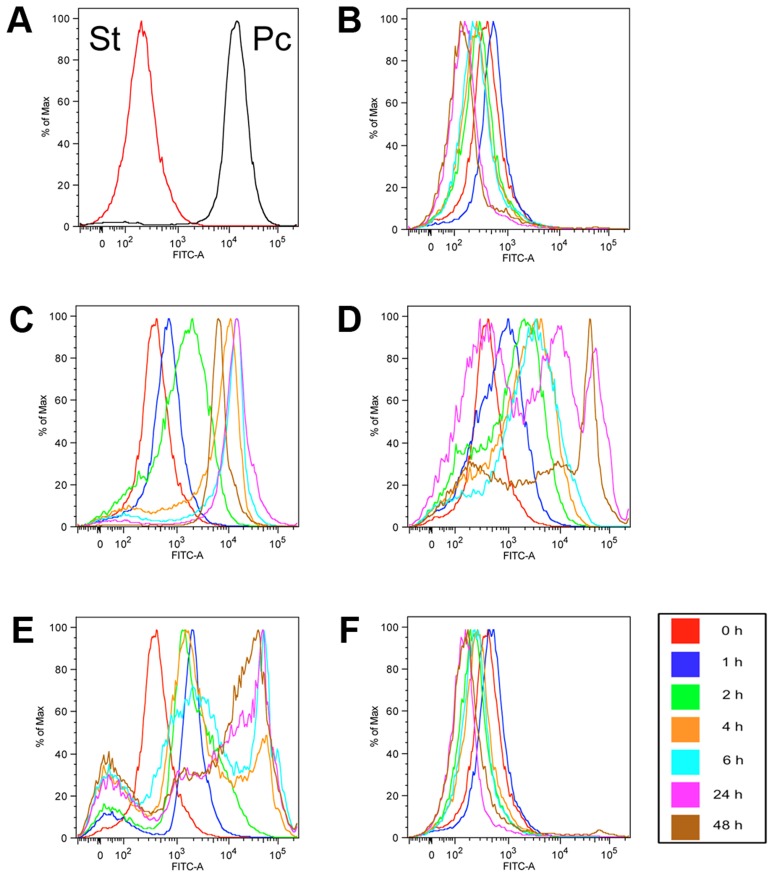
Evaluation of differentiation stimuli on stumpy forms. Flow cytometry traces of EP procyclin expression on AnTat 1.1 90:13 stumpy cells. Stumpy forms (St) and Procyclic forms (Pc) were used as negative and positive controls for the EP procyclin staining, respectively (**A**) and samples from untreated stumpy cell cultures were also collected at the same timepoints as cells exposed to the different differentiation stimuli (**B**). A weak reversible cold induced expression of EP procyclin is observed. To stimulate differentiation cells were treated with 6 mM *cis*-aconitate (**C**), mild acid (**D**), pronase (**E**) and phloretin (**F**). Samples were assayed at 0 h, 1 h, 2 h, 4 h, 6 h, 24 h or 48 h after exposure to each proposed trigger. The flow cytometry traces are representative of five independent experiments, each giving similar results.

**Figure 2:**
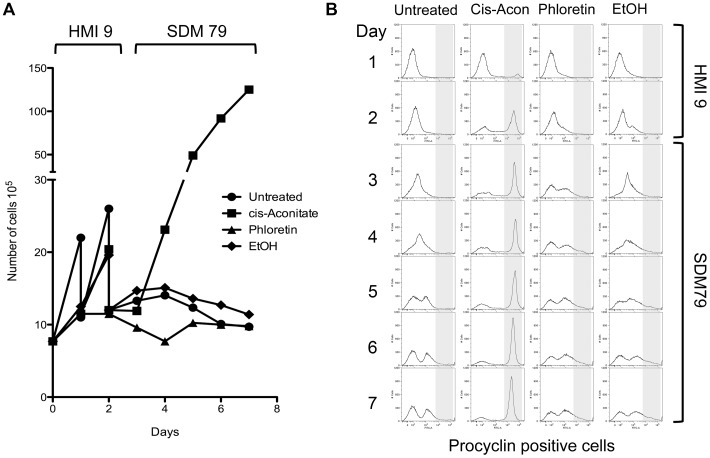
Phloretin is unable to induce differentiation of pleomorphic slender form cells to procyclic forms. *T. brucei* AnTat 1.1 90:13 cells (8×10^5^/ml) were cultured in HMI-9 at 37°C before the addition of 6 mM *cis*-aconitate, 100 µM phloretin or the same volume of 70% ethanol as a solvent control. After culture for 48 hours at 37°C, the cells were harvested, washed in phloretin-free procyclic medium (SDM-79), then inoculated into P-79 and incubated a further 5 days at 27°C. **A**. Phloretin treatment caused growth arrest within 24 hours in the pleomorphic slender cell cultures, with no outgrowth of procyclic cells being detected after transfer to phloretin-free procyclic medium. *Cis*-aconitate treated cells differentiated and proliferated as procyclic forms under the same culture conditions. **B**. The expression of the procyclic specific marker EP procyclin was monitored by flow cytometry for each culture. Whereas most *cis*-aconitate induced pleomorphic slender cells efficiently differentiated to procyclic forms within 48 hours, and expressed high levels of EP procyclin, phloretin treated cultures remained procyclin negative.

Figure 1 shows EP procyclin expression for stumpy or procyclic cells (Figure 1A) or stumpy cells incubated in the absence of any trigger (Figure 1B), demonstrating that stumpy cells do not express EP procyclin, although a reversible weak procyclin expression can be detected after incubation, reflecting cold-related expression of this marker [14]. However, when exposed to *cis*-aconitate (Figure 1C), mild acid (Figure 1D) or pronase (Figure 1E), the stumpy forms underwent effective differentiation into procyclic forms, with the expression of EP procyclin being evident after 2 hours, and becoming progressively stronger throughout the time course of the experiment. For the mild acid (Figure 1D) and pronase treatment (Figure 1E) there was some heterogeneity in procyclin expression due to stress associated killing or damage to some cells, this being absent for the CA treatment. In contrast, phloretin treated cells did not express EP procyclin (Figure 1F). To confirm that the phloretin treatment was effective, pleomorphic slender cells were incubated for 2 days in HMI-9 in the presence or absence of phloretin, or in the presence of *cis*-aconitate (Figure 2). Phloretin treatment arrested the growth of the treated cells within 24 hours, as expected if glucose uptake were prevented (Figure 2A). Nonetheless, these cells did not outgrow as differentiated forms when incubated in procyclic form medium nor did they express EP procyclin (Figure 2B), contrasting with CA treated cells. Furthermore, incubation of stumpy forms in a more physiologically relevant medium [32] containing low glucose (∼0.5 mM final concentration) did not stimulate their differentiation, unless *cis*-aconitate was also included (Figure S1 in Text S1). Hence, neither pleomorphic slender or stumpy forms differentiated in response to phloretin and stumpy cells were not stimulated by low glucose medium, demonstrating the glucose depletion is not an effective differentiation stimulus.

**Figure 3:**
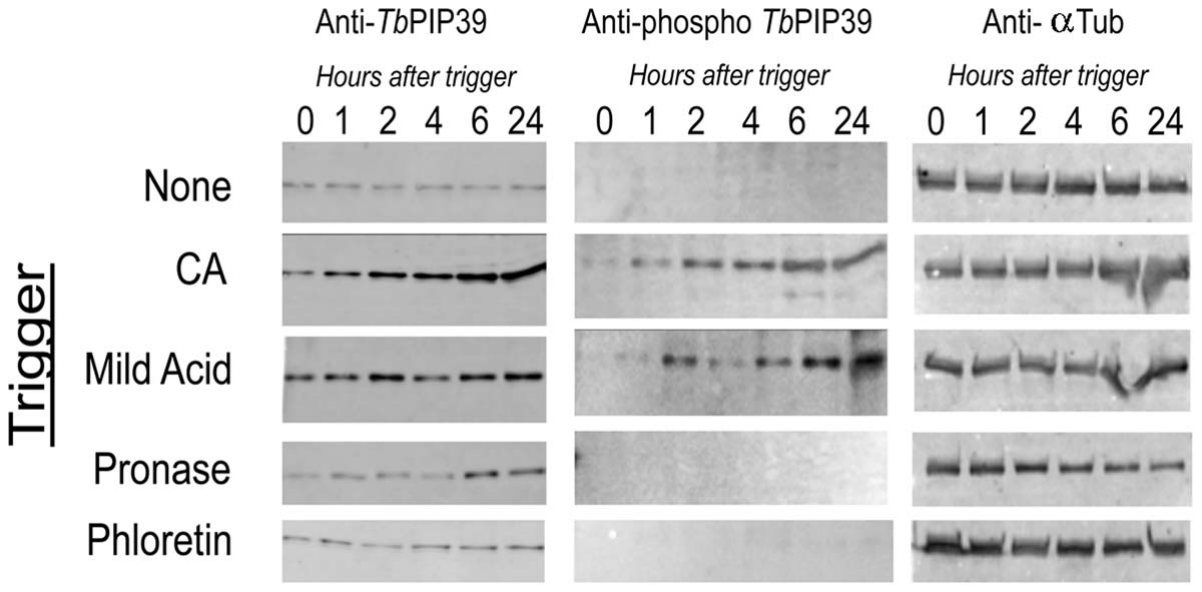
Differential *Tb*PIP39 phosphorylation in response to different differentiation signals. Stumpy cells were exposed to 6*cis*-aconitate (CA), mild acid (pH 5.5), pronase (4 units/ml) and phloretin (100 µM) and isolated protein samples were then reacted with a phospho-specific antibody recognising the sequence (ELDHWRTDEY*TK C) (anti-phospho *Tb*PIP39) (middle panels). The same blot was also reacted with an anti-*Tb*PIP39 polyclonal antibody (left hand panel) and with an antibody detecting trypanosome alpha tubulin as a loading control (right hand panels). Phosphorylated *Tb*PIP39 was observed with CA and mild acid treatment, but not pronase.

Having confirmed *cis*-aconitate, mild acid and pronase treatment as effective stimuli of differentiation, we monitored the phosphorylation of *Tb*PIP39 under each condition using a phospho-specific antibody directed against tyrosine 278 residue in the protein. This is the site of *Tb*PTP1 phosphatase activity [21], such that tyrosine 278 phosphorylation is diagnostic for the activity of this signal transduction pathway. Figure 3 shows the level of *Tb*PIP39, detected using a polyclonal antibody to the protein, and the level of phosphorylated *Tb*PIP39 detected using the tyrosine 278-phosphospecific antibody. As expected, untreated cells showed no evidence of *Tb*PIP39 phosphorylation, whereas the level of phosphorylated *Tb*PIP39 progressively increased throughout differentiation in the CA treated samples, with this being phosphorylated. Similarly, mild acid exposure generated phosphorylated *Tb*PIP39, indicating activity of the *Tb*PTP1/*Tb*PIP39 signalling pathway under that treatment regimen. Interestingly, however, no phosphorylated *Tb*PIP39 was detected after pronase treatment, despite the effective induction of differentiation in these cells as determined by EP procyclin expression (see Figure 1E, from the same experiment). As expected, exposure to phloretin did not induce *Tb*PIP39 phosphorylation.

These results indicated that CA and mild acid exposure of stumpy cells induces differentiation *via Tb*PTP1/*Tb*PIP39, whereas pronase treatment stimulates an alternative pathway that does not generate phosphorylated *Tb*PIP39. To test this hypothesis we exploited a pleomorphic *T. brucei* AnTat1.1 *Tb*PIP39 RNAi cell line that generates effective and inducible *Tb*PIP39 depletion in stumpy forms [21]. Our prediction was that *Tb*PIP39 depletion would reduce the efficiency of differentiation for those stimuli (CA, mild acid) that operate via this signalling pathway, whereas there would be no effect upon pronase treatment. Figure S2 in Text S1 shows Western blots indicating the level of *Tb*PIP39 in stumpy forms in which RNAi against the *Tb*PIP39 transcript had been induced, or not, by provision of doxycycline to the drinking water of infected mice. In each case there was a clear evidence of *Tb*PIP39 depletion in those samples where RNAi was induced in the host animals this being particularly evident as the cells underwent differentiation, whereupon the levels of *Tb*PIP39 normally increase (see Figure 3 and [21]).

**Figure 4:**
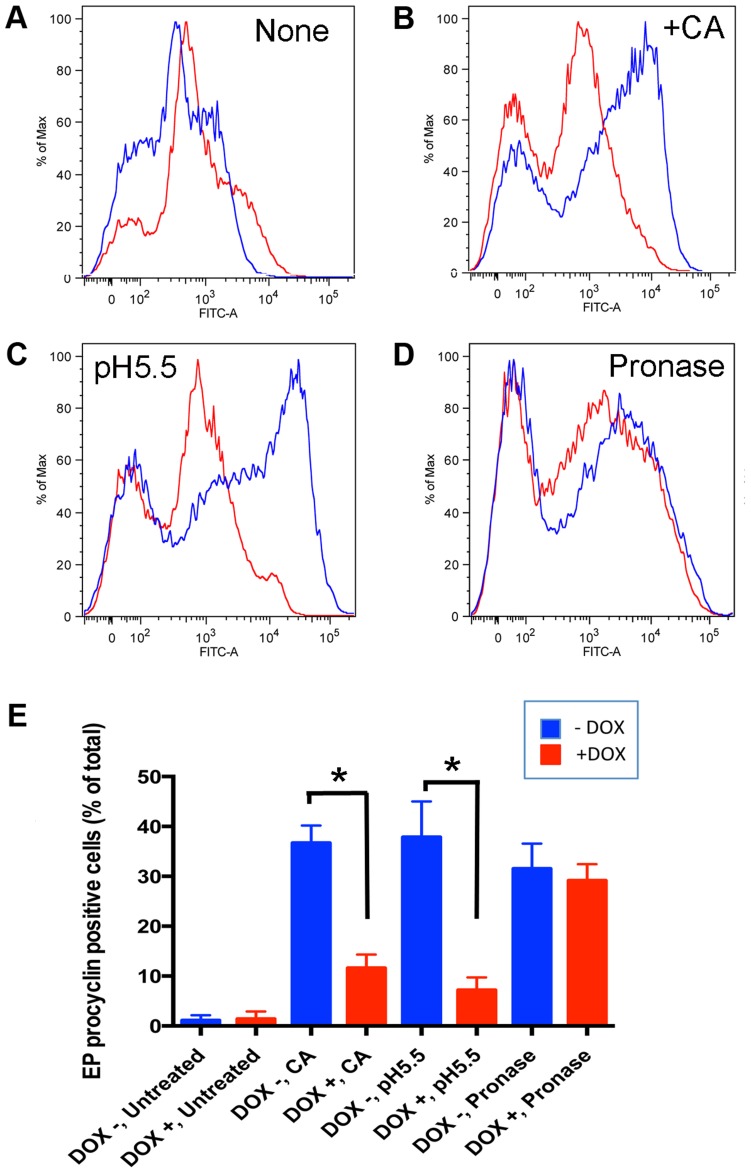
RNAi mediated ablation of *Tb*PIP39 reduces the differentiation efficiency of cells treated with cis-aconitate and mild acid, but not pronase. *Tb*PIP39-RNAi stumpy cells, either uninduced (−Dox) or induced (+Dox) with doxycycline *in vivo* were harvested, inoculated into HMI-9 at 37°C before the exposure to ‘no treatment’ (**A**), 6 mM *cis*-aconitate (CA) (**B**), mild acid (pH 5.5) (**C**) or pronase (4 units/ml) (**D**). Samples of the untreated and treated (**B–D**) cultures were prepared at 0 h and 4 h and differentiation analysed by the expression of the differentiation marker EP procyclin. The treated cells showed a population of undifferentiated trypanosomes (left hand peak) representing slender cells in the population and dead or damaged cells, particularly after pronase treatment. Panel E shows histograms of the percentage of EP procyclin expressing cells at 4 h after exposure to the different treatments, the results being the mean and standard deviation of 4 experiments (3 for pronase treatment). Asterisks indicate significance (p<0.001). *Tb*PIP39 depletion resulted in reduced differentiation for the *cis*-aconitate and mild acid treated samples, but not those exposed to pronase.

To assess the differentiation efficiency of the cells, EP procyclin expression was monitored at 4 h after exposure to each trigger, before the outgrowth of differentiated cells and the consequences of the low levels of *Tb*PIP39 that remain after RNAi induction complicate the analysis [21]. Figure 4A shows that untreated cells did not differentiate, with only the weak cold-related expression of procyclin detected, as also seen in Figure 1B. When triggered with CA (Figure 4B) *Tb*PIP39 RNAi reduced the differentiation efficiency, and a similar response was also observed when cells were treated with mild acid (Figure 4C). Upon pronase treatment there was a population of unstained cells, representing undifferentiated slender cells (also seen in Figures 4A–C) and dead or damaged cells generated by the pronase treatment. However, when analysing EP procyclin expressing cells, either induced or uninduced to deplete *Tb*PIP39, approximately equivalent differentiation efficiency was observed (Figure 4D), consistent with *Tb*PIP39-independent differentiation. The same outcome was observed in four independent experiments, with *Tb*PIP39 depletion reducing differentiation triggered by CA (p<0.001; general linear mixed model) and mild acid (p<0.001) but not pronase (p = 0.4270) (Figure 4E). We conclude that two independent pathways can stimulate differentiation, one initiated by CA or mild acid, that acts *via Tb*PIP39 phosphorylation, and one, stimulated by pronase, that does not.

### Differentiation triggers operate independently in the tsetse midgut

**Figure 5:**
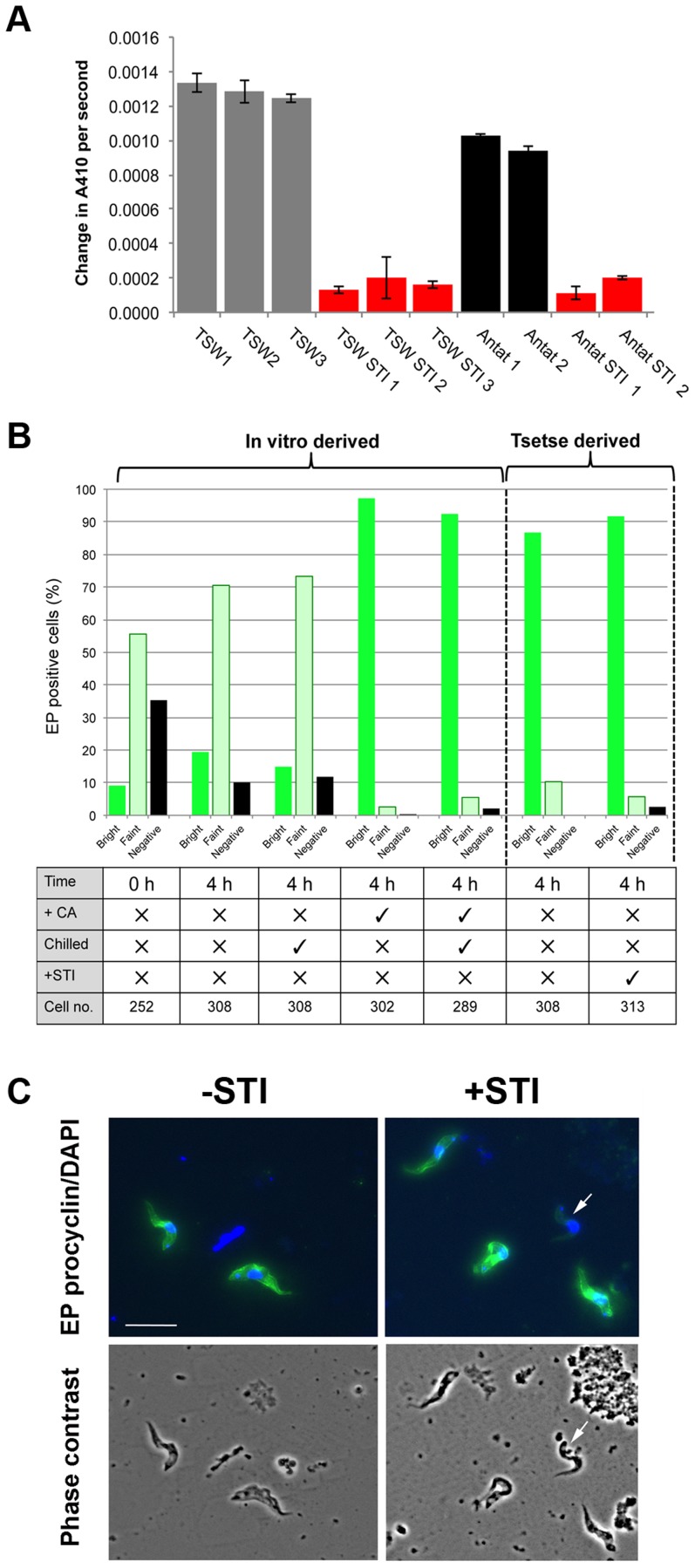
Protease activity is not necessary for differentiation in tsetse flies. **A**. Protease activity (against Chromozym Try substrate) in midgut extracts from tsetse flies fed horse serum containing *T. brucei* TSW strain trypanosomes, or *T. brucei* AnTat.1.1, either in the presence or absence of Soybean trypsin inhibitor (STI). Each bar represents the average protease activity ±SEM of 10 midguts. **B**. EP procyclin expression of stumpy trypanosomes incubated *in vitro* in HMI-9 or derived from tsetse midguts 4 h after feeding with horse serum containing the parasites, with or without soybean trypsin inhibitor (±STI). Stumpy cells were analysed at 0 hours, or after 4 hours *in vitro* in the presence or absence of *cis*-aconitate (‘CA’). To control for the chilling on ice associated with tsetse midgut dissection, some *in vitro* derived samples were also chilled for 1 hour at 4°C, particular attention being taken to precisely mimic the processing of tsetse derived samples. Scoring was carried out by immunofluorescence, with EP procyclin staining scored as negative, faint or bright in each case. In all cases samples were processed for immunofluorescence in parallel and imaged under identical settings. **C**. Representative images of trypanosomes derived from tsetse midguts 4 h after feeding either in the presence or absence of soybean trypsin inhibitor. Cells were stained with antibody to EP procyclin (Green) and DAPI (Blue). A cell categorised as ‘faint labelling is arrowed. The lower images show phase contrast images of the equivalent fields. Bar = 20 µm.

We analysed the relevance of the different signalling pathways *in vivo* by assaying tsetse infections when protease activity was blocked using inhibitors. Thus, batches of tsetse flies were fed with trypanosomes in horse serum either in the presence or absence of 1 mg/ml of soybean trypsin inhibitor (STI), a treatment predicted to block the insect midgut trypsin-like activities that comprise a major digestive component of the tsetse midgut [33] and which have been reported to stimulate differentiation *in vivo* [34], [35] and *in vitro* [26]. Confirming the efficacy of inhibition, an analysis of 30 tsetse extracts revealed that midgut trypsin/chymotrypsin activity was reduced 84%–100% in three replicate experiments (P<0.01), irrespective of whether trypanosomes were included in the serum meal (Figure 5A). Stumpy forms were then fed to flies in horse serum in the presence or absence of STI and differentiated cells analysed in extracted midguts 4 hours after feeding. Replicate stumpy samples were also exposed to CA in culture and particular care was taken to ensure that tsetse fed and *in vitro* prepared samples were processed under identical conditions in order to eliminate effects attributable to cold-induced procyclin expression [14]. In each case, since neither flow cytometry nor automated fluorescence analysis proved possible due to the debris present in the tsetse midgut extracts, we analysed EP procyclin expression using a careful visual scoring system, categorising the labelling of cells as ‘bright’ (representing a homogenous, bright, EP signal detected on the whole cell), ‘faint’ (a faint and/or non-homogeneous EP signal detected on the whole cell, with a brighter flagellum and/or uneven, punctuated staining pattern) or ‘negative’ (no EP signal detected). Figure 5B demonstrates that under these conditions, EP procyclin was strongly expressed on ∼90% of cells from tsetse midguts regardless of the presence or absence of protease inhibitor, matching *in vitro* differentiation in the presence of CA (representative images are shown in Figure 5C and Figure S3 in Text S1). Untreated samples showed only faint EP procyclin expression associated with cold shock during sample processing. Furthermore, similar numbers of cells were observed in STI treated and untreated midgut extracts, eliminating the possibility that only those few cells able to differentiate had survived in the midguts of the flies fed trypsin inhibitor. We conclude that blocking protease activity in the tsetse midgut does not prevent differentiation.

**Figure 6:**
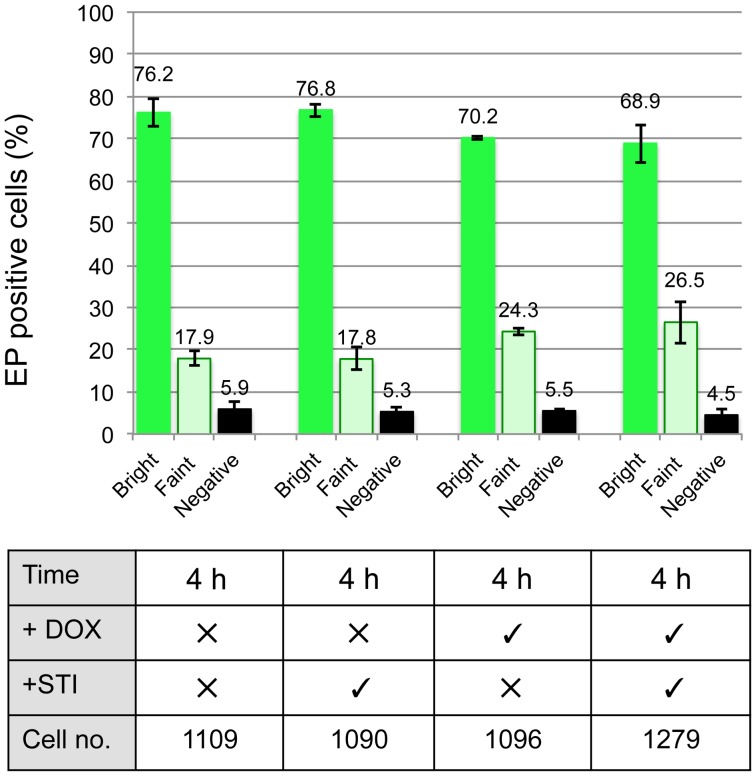
Differentiation trypanosomes in tsetse flies is reduced upon *Tb*PIP39 RNAi. Stumpy form *Tb*PIP39 RNAi cells were fed to tsetse flies in the presence of absence of STI. After 4 h midguts were extracted and the percentage of procyclin expressers determined by immunofluorescence microscopy. The data represents an analysis from 3 independent infections with the total number of cells scored being shown in each case.

Having demonstrated that inhibiting trypsin activity in the bloodmeal did not prevent the differentiation of stumpy forms, we repeated the analysis using the pleomorphic *Tb*PIP39 RNAi line to determine the influence of inhibiting the *Tb*PIP39 signalling pathway alone, or in combination with trypsin inhibition. Specifically, parasites were grown in mice provided, or not, with doxycycline in their drinking water to induce *Tb*PIP39 gene silencing during the infection. Aliquots of the resulting cells were then fed to tsetse flies either in the presence or absence STI, such that in individual analyses either or both proteolytic activity and cellular *Tb*PIP39 was depleted. Gut extracts were isolated 4 h after feeding, and the differentiation of the midgut parasites analysed by immunofluorescence for the expression of EP procyclin as before, with at least 1000 cells being scored for each sample from a total of 3 replicate experiments (Figure 6). Confirming the data in Figure 5, trypsin inhibition alone did not inhibit differentiation (p = 0.5706; Wilcoxon Mann Whitney Rank sum test). However, with *Tb*PIP39 depletion, the percentage of weakly stained cells increased from approximately 17.9% (17.9%, 17.8% + or – STI, respectively) for the uninduced samples to 25.4% (24.3%, 26.5% + or – STI, respectively), and overall differentiation was reduced, weakly but significantly (p = 0.0073), even when a Bonferroni correction was used to account for the number of tests (thereby setting the significance threshold at 0.008). Interestingly, however, when both RNAi was induced and proteases were inhibited, the efficiency of differentiation was more significantly reduced (p<0.0009) with respect to untreated samples. Although the observed effects were expected to be minor, given the transient inhibition of differentiation upon *Tb*PIP39 depletion *in vitro*, these analyses indicated that inhibition of differentiation is observed *in vivo* upon *Tb*PIP39 depletion, with a minor contribution when trypsin activity is also inhibited.

### The interaction between TbPTP1 and TbPIP39 is citrate dependent

The *in vivo* experiments supported the function of the *Tb*PTP1/*Tb*PIP39 pathway in differentiation, but the technical limitations with these studies highlighted that more detailed analysis of these signalling events required *in vitro* analysis. Therefore, we sought to gain mechanistic understanding of the interaction between *Tb*PTP1 and *Tb*PIP39 using biochemical methods.

**Figure 7:**
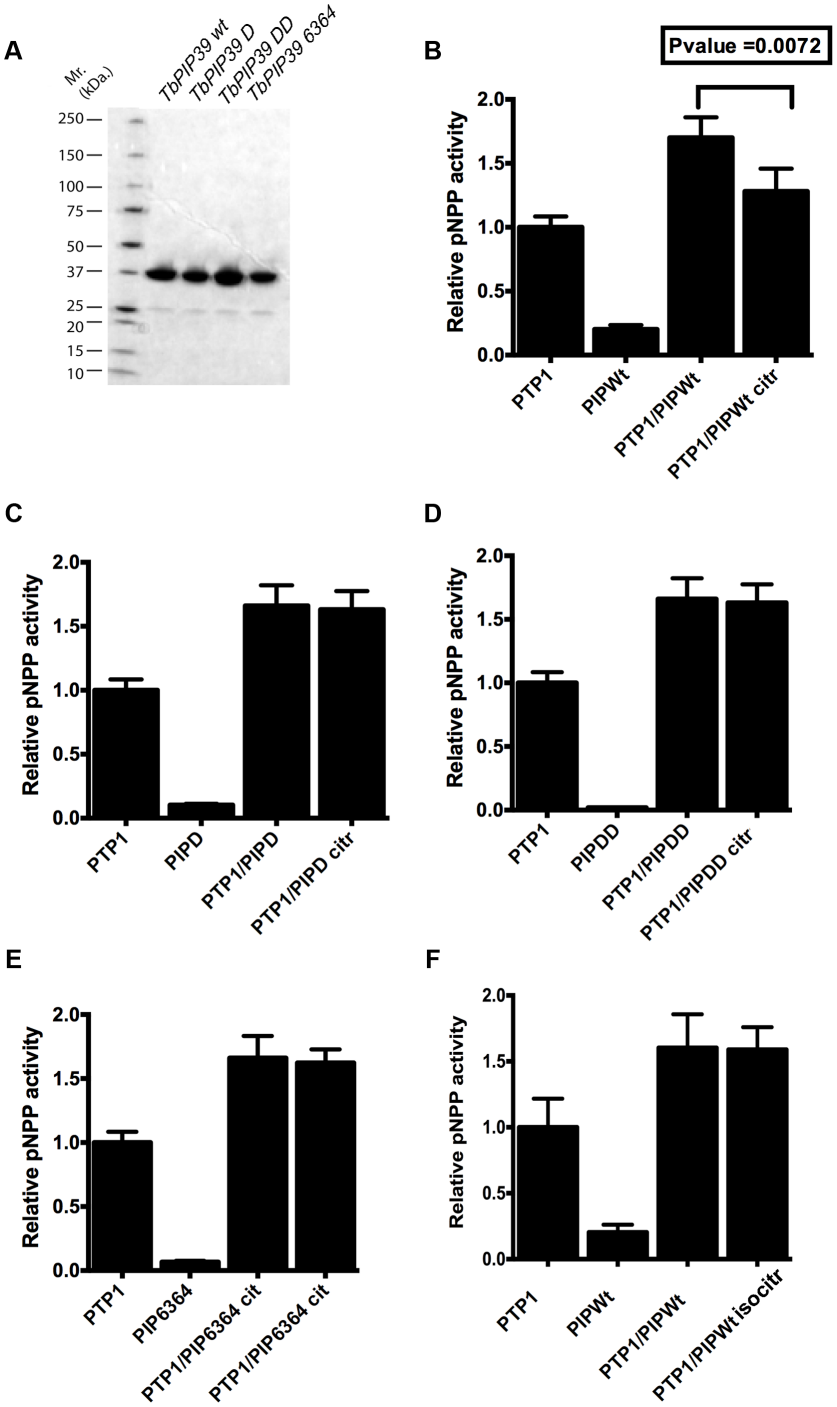
*Tb*PIP39 mutants promote *Tb*PTP1 activity but lack citrate responsiveness. (**A**) Recombinant proteins of wild type and mutant *Tb*PIP39 used for biochemical and biophysical analyses. **(B)–(F)** The protein phosphatase activity of *Tb*PTP1 (0.1 µg) and various forms of *Tb*PTP1 (1 µg of wild type, and the citrate binding mutants D, DD and 6364) were each measured. The combined pNPP activity of *Tb*PTP1 and *Tb*PIP39 were assayed in the absence and presence of citrate (2 mM) or, for the wild type *Tb*PIP39, in the presence of 2 mM isocitrate, which is not a differentiation trigger (Panel F). In each case, *Tb*PTP1 pNPP activity was normalised to 1.0 and all other pNPP activities were expressed relative to that. The error bars represent SD values of 5 independent triplicate assays. Only wild type *Tb*PIP39 exhibited citrate-dependent inhibition of *Tb*PTP1/*Tb*PIP39 activity.

Initially, we investigated whether residues in *Tb*PIP39 predicted from homology modelling to comprise part of a citrate-binding pocket [21] (Figure S4 in Text S1) would influence the regulatory cross talk between *Tb*PIP39 and *Tb*PTP1, whereby *Tb*PIP39 activates *Tb*PTP1, unless in the presence of citrate [21]. To analyse this, recombinant *Tb*PIP39 was generated in which either the aspartic acid at position 57 was mutated to alanine (generating *DxAxT*; “PIP39 D mutant”), both aspartic acid residues in the *DxDxT* motif were mutated (generating *AxAxT*; “PIP39 DD mutant”) or the TV motif at position 63 and 64 was mutated to *AA* (“PIP39 6364 mutant”) (Figure S4 in Text S1; the respective recombinant proteins are shown in Figure 7A). The residues at position 57, 63 and 64 are each predicted to be involved in citrate binding whereas mutating both aspartic acid residues is known to render the enzyme catalytically inactive [21], [36]. The wild type (Figure 7B) and *Tb*PIP39 mutants (Figure 7C–E) were each then tested for their ability to enhance *Tb*PTP1 activity and the sensitivity of this to citrate inhibition. Figure 7 demonstrates that each of the *Tb*PIP39 mutants (*Tb*PIP39 D, *Tb*PIP39 DD, *Tb*PIP39 6364) enhanced the activity of *Tb*PTP1 at equivalent levels to the wild type *Tb*PIP39, regardless of their individual activity. However, this was not sensitive to the presence of citrate for any of the mutants, contrasting with the wild type protein where there was a citrate-dependent decrease in *Tb*PTP1 activity (P<0.0072). Unlike citrate, isocitrate (which does not act as a differentiation trigger) did not generate a decrease in activity when wild type *Tb*PIP39 was used (Figure 7F). This demonstrated that the regulatory cross talk between *Tb*PTP1 and *Tb*PIP39 depended upon the integrity of the citrate binding residues in *Tb*PIP39.

**Figure 8:**
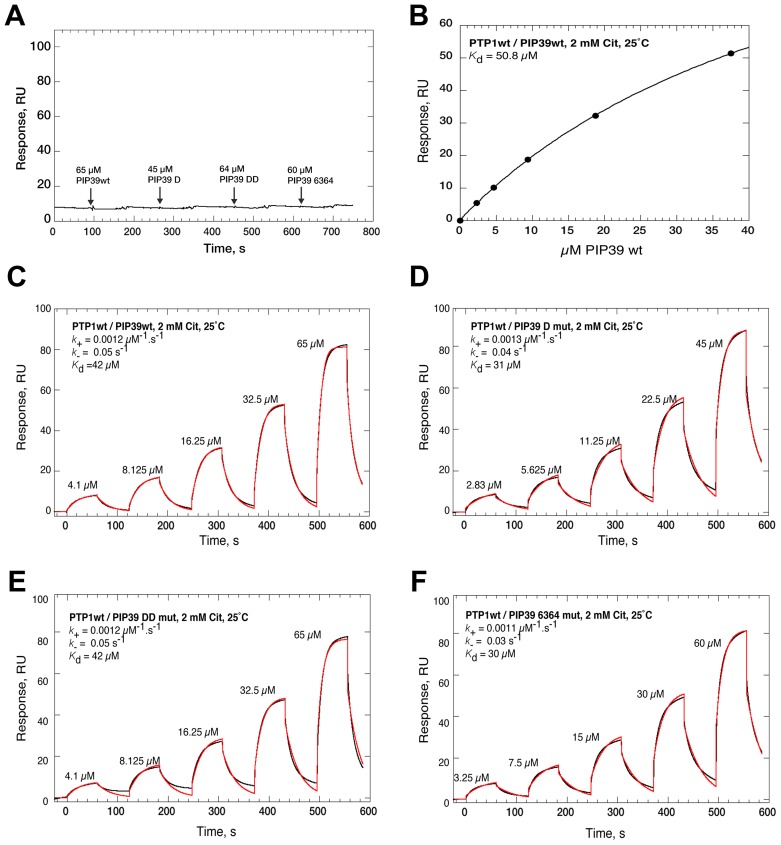
Characterisation of the interaction of *Tb*PTP1 with *Tb*PIP39 (wild type and mutants), in the presence or absence of citrate using BIAcore T200. **A**. Representative double reference corrected single cycle kinetic titration SPR binding curves (black), monitored on a surface of covalently stabilized His-PTP1wt, for *Tb*PIP39wt (65 µM), *Tb*PIP39 D mutant (45 µM), *Tb*PIP39 DD mutant (64 µM) or *Tb*PIP39 6364 mutant (60 µM) at 25°C in 10 mM HEPES, pH 7.4; 150 mM NaCl; 0.05% P20; 2 mM DTT; 20 mM MgCl2 in the absence of citrate. **B**. Steady state analysis of the interaction between *Tb*PTP1 and *Tb*PIP39 by surface plasmon resonance. The assay was run in 10 mM HEPES, pH 7.4; 150 mM NaCl; 0.05% P20; 2 mM DTT; 20 mM MgCl2; 2 mM citrate. A concentration dependent interaction between *Tb*PTP1wt and *Tb*PIP39wt was seen. All fits were to a 1∶1 binding model, with mass transport considerations. Kd is ∼40–50 uM. **C–D** Representative double reference corrected single cycle kinetic titration SPR binding curves (black), monitored on a surface of covalently stabilized His-PTP1wt, for *Tb*PIP39wt (C), *Tb*PIP39 D mutant (D), *Tb*PIP39 DD mutant (E) or *Tb*PIP39 6364 mutant (F) at 25°C in 10 mM HEPES, pH 7.4; 150 mM NaCl; 0.05% P20; 2 mM DTT; 20 mM MgCl_2_ with 2 mM citrate. In each case, a three-fold dilution series of *Tb*PIP39 (wild type and mutants) was injected over the surface, at 30 µl.min-1 and the apparent on- and off-rate constants were by globally fitting (red) a 1∶1 kinetic binding model, with mass transport considerations, to the sensorgrams using the analysis software (v1.02, GE Healthcare) supplied with the instrument.

Having examined regulatory interaction between the molecules we examined their biophysical interactions in the presence or absence of citrate using surface plasmon resonance. Specifically, *Tb*PTP1 protein was immobilised and covalently stabilised on a nitrilotriacetic acid (NTA) chip and then interactions with wild type or mutant *Tb*PIP39 tested in the presence or absence of citrate (Figure 8). In the absence of citrate, no interaction between *Tb*PTP1 and *Tb*PIP39 (wild type or any of the *Tb*PIP39 mutants) was observed (Figure 8A, Figure S5 in Text S1). In contrast, wild type *Tb*PIP39 showed interaction with *Tb*PTP1 in the presence of citrate, consistent with a 1∶1 binding stoichiometry, the predicted half-life for the complex being around 10 seconds (on rate: 0.015 µM-1.s-1; off rate: 0.068 s-1) (Figure 8B, 8C, Figure S5 in Text S1). Interestingly, when the mutant forms of *Tb*PIP39 were tested in combination with *Tb*PTP1 by surface plasmon resonance, each also showed a citrate-dependent interaction with *Tb*PTP1, matching that of the wild type *Tb*PIP39 (Figure 8D–F). Moreover, isocitrate, which does not stimulate differentiation [26], [37], also promoted the interaction between *Tb*PTP1 and wild type *Tb*PIP39 (on rate: 0.014 µM-1.s-1; off rate: 0.064 s-1) indicating that the interaction is not contributing to the specific differentiation stimulus (Figure S5 in Text S1). When analysed by isothermal calorimetry neither the wild type nor any of the *Tb*PIP39 mutant proteins was able to bind citrate independently of *Tb*PTP1 (Figure S6 in Text S1). Overall, these experiments indicated that individual mutation of specific residues in *Tb*PIP39 predicted to be involved in citrate binding did not prevent the citrate-dependent interaction of *Tb*PIP39 with *Tb*PTP1 detected by SPR and that this interaction was also generated by isocitrate, which is not a differentiation trigger.

In conclusion, the ability of citrate to reduce the phosphatase activity of *Tb*PTP1/*Tb*PIP39 was lost upon mutation of the predicted citrate-binding residues in *Tb*PIP39. Nonetheless, regardless of the integrity of these residues, these molecules continue to interact in a citrate (or isocitrate) dependent-manner. Based on these analyses we propose that citrate causes *Tb*PIP39 and *Tb*PTP1 to bind to one another, at least *in vitro*, but that citrate also independently blocks the ability of *Tb*PIP39 to enhance *Tb*PTP1 activity. Hence, *in vivo*, the transport of citrate in the bloodmeal by PAD proteins would stimulate the initiation of parasite differentiation through the co-ordinated inhibition of *Tb*PTP1 and activation of *Tb*PIP39.

## Discussion

When ingested in a tsetse fly bloodmeal trypanosomes rapidly initiate differentiation in order to adapt to their new environment. Our earlier studies have demonstrated that the CCA differentiation signal is transduced via PAD proteins through a phosphatase-signalling cascade, whereby *Tb*PTP1 is inactivated and *Tb*PIP39 becomes phosphorylated and activated [20]–[22]. Here we have used these components to investigate the molecular basis of differentiation initiated by CA and to demonstrate that independent signalling pathways can operate to stimulate development.

Firstly, the availability of phospho-specific *Tb*PIP39 antibodies and pleomorphic RNAi lines targeting *Tb*PIP39 allowed the relationship between different differentiation signalling pathways to be investigated. This demonstrated that CA and mild acid are triggers that both result in the phosphorylation of *Tb*PIP39 on tyrosine 278, with differentiation via these signals being inhibited by RNAi against *Tb*PIP39. In contrast, pronase stimulates differentiation effectively but this does not generate phosphorylated *Tb*PIP39 nor does RNAi against this molecule inhibit it. In mammals, protease activated small G protein coupled receptors, PARs, operate to regulate signalling events [38], but conventional G-protein signalling is missing in trypanosomes [39]. Hence, although cleavage-mediated activation might also contribute to the initiation of differentiation in trypanosomes, the molecule responsible is likely to be novel, potentially at the cell surface or within the flagellar pocket membrane.

**Figure 9:**
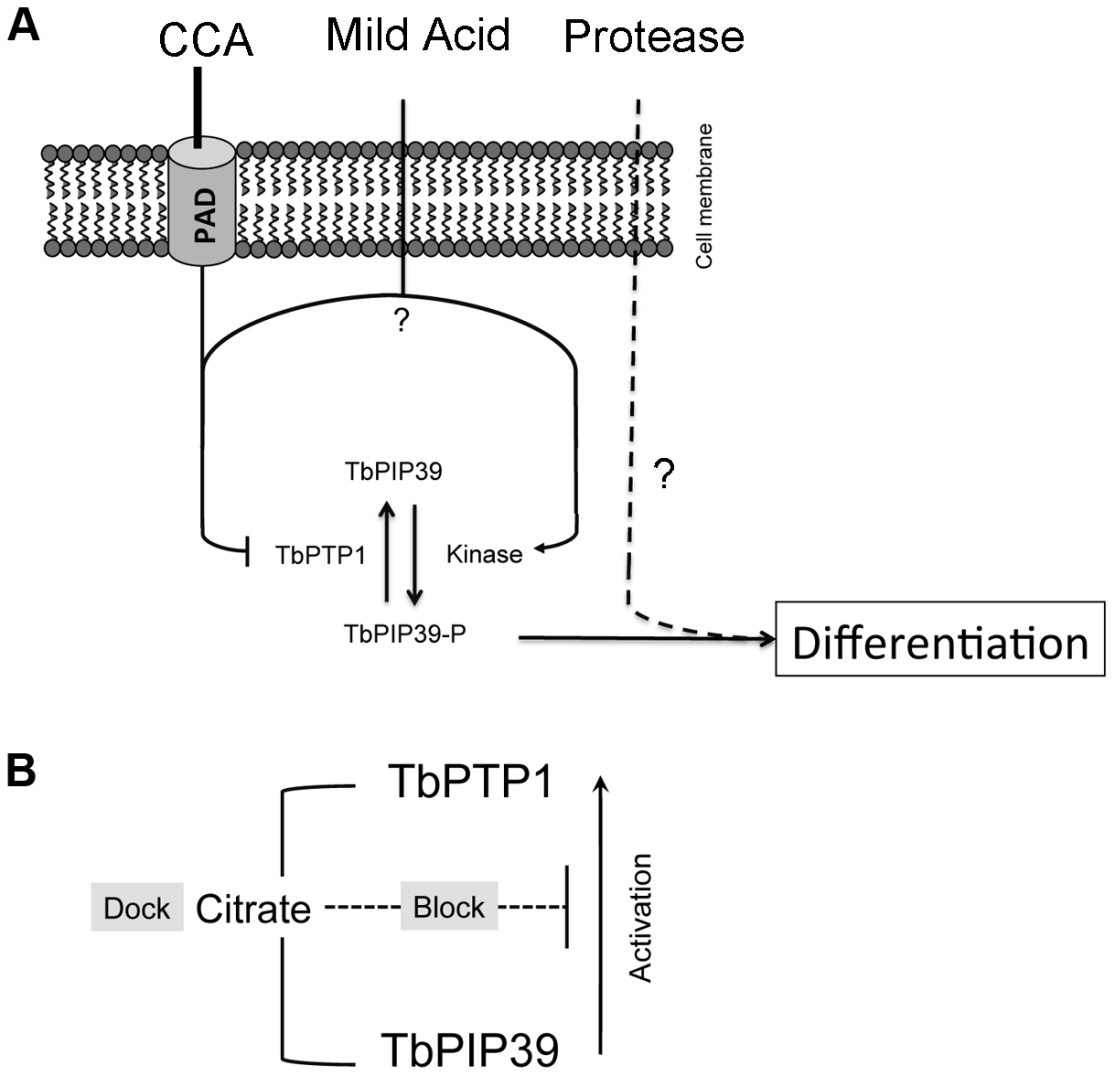
Model for the initiation of differentiation via different triggers. **A**. CCA and mild acid result in an increase in *Tb*PIP39 phosphorylation that stimulates differentiation. For CCA, this is mediated via the transport of citrate/*cis*-aconitate *via* surface PAD proteins. Mild acid may inhibit *Tb*PTP1, and/or stimulate the activity of the kinase that phosphorylates *Tb*PIP39. Pronase also stimulates differentiation, independently of *Tb*PTP1/*Tb*PIP39. **B**. Mechanistically, CCA stimulates both the interaction between *Tb*PTP1 and *Tb*PIP39 (Dock), and inhibition of the stimulatory effect of *Tb*PIP39 on *Tb*PTP1 (Block).

Our findings reveal the presence of independent differentiation signalling pathways in trypanosomes (Figure 9A), with activation by different stimuli converging downstream of *Tb*PIP39. Supportive of the existence of independent signalling pathways, reporter assays for the activation of EP procyclin expression in bloodstream forms have demonstrated enhanced expression when both CA and proteases were used compared with either stimulus alone [26]. Our *in vivo* experiments supported this observation, since the depletion of *Tb*PIP39 by RNAi reduced differentiation, the significance of this being increased in the presence of protease inhibitors. Although the reduction of *Tb*PIP39 by RNAi is incomplete, significantly reducing the magnitude of the effect observed *in vivo*, trypsin/chymotrypsin-type protease inhibition was almost complete when monitored in the midgut of treated flies. Hence, although trypsin activities have been reported [33] and proposed to be involved in the transformation of trypanosomes *in vivo* [34], [35] and *in vitro* [26], our experiments demonstrate that inhibiting this protease activity alone does not prevent the initiation of parasite differentiation in tsetse flies. Unfortunately, a complementary analysis of differentiation in the complete absence of *Tb*PIP39 via a gene knockout and add-back approach is not possible in parasite lines capable of generating stumpy forms.

In contrast to the above reported triggers we did not observe any induction of differentiation for either bloodstream stumpy forms or bloodstream slender forms when parasites were exposed to phloretin or low glucose conditions. Phloretin blocks glucose uptake by the trypanosome and has been reported to induce the expression of a subset of procyclic specific transcripts in monomorphic bloodstream forms [29]. An effect of glucose depletion has also been reported earlier, whereby glucose-depleted medium generated the outgrowth of differentiated procyclic forms from a monomorphic cell culture [28]. Although these findings seem consistent with the rapid loss of glucose from the tsetse blood meal, they may also reflect a monomorphic cell line specific response, or the outgrowth of a small, differentiated subpopulation when cells are subcultured into procyclic form media. Alternatively, phloretin may induce transformation of monomorphic slender forms toward more stumpy-like forms, since stumpy forms elevate the expression of some procyclic form transcripts including procyclins in preparation for differentiation in the tsetse fly or *in vitro* [29]. Nonetheless, overall our results eliminate glucose depletion alone as an efficient differentiation trigger for transmissible stumpy forms, making the physiological relevance of this trigger questionable.

Through biochemical and biophysicial analyses, our experiments also revealed that *Tb*PTP1 and *Tb*PIP39 interact in a citrate or isocitrate dependent manner, this being retained even when individual residues proposed to be important in citrate binding within *Tb*PIP39 are mutated. However, mutation of these residues prevented the citrate-dependent reduction of *Tb*PTP1/*Tb*PIP39 activity. These results contrast with a model where citrate binding to *Tb*PIP39 would prevent its interaction with *Tb*PTP1 [21], [22], potentially through steric hindrance within the catalytic region. Instead, our results show that the presence of citrate can allow *Tb*PTP1 and *Tb*PIP39 to physically engage, at least *in vitro*, potentially in a substrate trapping type interaction although this is not specific for citrate or the predicted citrate binding capacity of *Tb*PIP39. Hence, in a bloodmeal, *Tb*PTP1 could be sequestered by interaction with *Tb*PIP39 such that excess unbound *Tb*PIP39 could become stably phosphorylated by its, as yet uncharacterised, kinase thereby promoting differentiation. Furthermore, these experiments revealed that the predicted citrate binding residues in *Tb*PIP39 are required for the regulatory cross talk between *Tb*PIP39 and *Tb*PTP1 but not their specific interaction, suggesting that citrate has distinct ‘dock’ and ‘block’ activities on the *Tb*PIP39/*Tb*PTP1 complex, with only the ‘blocking’ function being specific for citrate (Figure 9B).

The integration of the combined signals of temperature reduction, CCA reception and protease attack of the parasite surface could ensure a strong differentiation response. Given their sensitivity to citrate/*cis*-aconitate and resistance to protease attack [40], these responses will be limited to the transmissible stumpy form, with slender cells in the blood meal being rapidly killed. The role of multiple signal inputs that converge to drive differentiation is characteristic of several developmental systems including in fungi [41], cell type differentiation in *Drosophila* development [42], [43] and in mammalian bone formation [44], as well as in arthropod-borne parasites [5], [8]. In parasites, this stringent control would ensure that the initiation of an irreversible developmental programme occurs only under the correct environmental conditions, avoiding the risk of initiating an inappropriate and lethal differentiation response whilst still in the mammalian host.

## Materials and Methods

### Ethics statement

Animal experiments in this work were carried out in accordance with the local ethical approval requirements of the University of Edinburgh and the UK Home Office Animal (Scientific Procedures) Act (1986) under licence number 60/4373.

### Parasite growth and transfection

Bloodstream and procyclic form trypanosomes were cultured *in vitro* in HMI-9 [45] medium or SDM-79 [46] medium respectively. *T. brucei* AnTat1.1 slender and stumpy parasites were obtained 3 and 6 days post infection, respectively, and purified by DEAE chromatography. *Tb*PIP39 RNAi lines were described in [21].

For the initiation of differentiation the following conditions were used:

#### cis–aconitate

6 mM cis-aconitate provided in HMI-9 medium.

#### Mild acid

Stumpy cells were incubated in HMI-9 media, whose pH was adjusted to 5.5. After 2 hours incubation at 37 °C, 5% CO_2_ the cells were collected, washed twice in fresh HMI-9 media and transferred to 27°C [24].

#### Pronase

Stumpy cells (2–5×10^6^ cells/ml) were resuspended in PSG buffer containing 4 u/ml pronase (Biochemika) and incubated for 10 minutes at 25°C [25], [26]. After incubation, the cells were collected by centrifugation, washed twice in filtered PSG and resuspended in fresh HMI-9 media and transferred to 27°C.

#### Phloretin

*T. brucei* AnTat 1.1 90:13 cells (8×10^5^/ml cell density) were treated with 100 µM phloretin (Biomol) and cultured in HMI-9 at 37°C for 48 hours in a CO_2_ incubator [29]. After two days, the cells were harvested and following two washes in phloretin-free procyclic form medium (SDM-79), they were inoculated into fresh SDM-79 and incubated for a further 5 days at 27°C. Cell numbers were monitored daily using a Beckman Coulter Z2 particle counter.

### DNA cloning and protein expression and analysis

Recoded wild type (wt) and ExExT (DD) synthetic pHD451*Tb*PIP39 constructs [21]were used to produce mutants predicted to have reduced ability to bind citrate (Figure S4 in Text S1). A commercial site-directed mutagenesis kit (Stratagene) was used with the mutagenesis Primer 1 and Primer 2 (Table S1) to produce *Tb*PIP39D57A (*Tb*PIP39D) and Primer 3 and Primer 4 (Table S1) to produce t63A v64A (*Tb*PIP39 6364). Each of these pHD451*Tb*PIP39 constructs (*Tb*PIP39wt, *Tb*PIP39D, *Tb*PIP39 6364 and *Tb*PIP39DD) were reamplified with recoded *Tb*PIP39 specific Primer 5 and Primer 6 (Table S1) and integrated into the pGEX4T1 (GE Healthcare Life sciences) protein expression vector for recombinant protein production. Expression and purification of His tagged *Tb*PTP1 and GST tagged *Tb*PIP39 (*Tb*PIP39wt, *Tb*PIP39D, *Tb*PIP39 6364 and *Tb*PIP39DD) were performed as described in [21]. Phosphatase activity was measured by monitoring the *Tb*PTP1 (0.01–1 µg) catalyzed hydrolysis of pNPP to *p*-nitrophenol [20].

Methods for protein expression and purification for biophysical analyses are described in the Supplementary data.

### Surface plasmon resonance (SPR) equipment and reagents

SPR measurements were performed on a BIAcore T200 instrument (GE Healthcare). Ni^2+^-nitrilotriacetic acid (NTA) sensor chips, 1-ethyl-3- (3-diaminopropyl) carbodiimide hydrochloride (EDC) and *N*-hydroxysuccinimide (NHS) were purchased from GE Healthcare.

### Immobilization and covalent stabilization *Tb*PTP1

Pure His-PTP1 was immobilized and covalently stabilized on an NTA sensor chip. Briefly, following Ni^2+^ priming (60 s of 500 µM NiSO4 in 10 mM HEPES, pH 7.5; 150 mM NaCl; 0.05% P20; 50 µM EDTA; 20 mM MgCl2, at 5 µl.min^−1^) dextran surface carboxylate groups were activated by injection of 20 µl of 0.2 M EDC; 50 mM NHS at 5 µl.min^−1^. Protein (between 10 nM and 100 nM) in 10 mM HEPES, pH 7.5; 150 mM NaCl; 0.05% P20; 50 µM EDTA; 20 mM MgCl_2_ was captured and covalently stabilized on the surface to between 100 and 300 RU by injection at 30 µl.min^−1^. Following attainment of the desired RU signal a brief injection of 10 mM HEPES, pH 7.5; 150 mM NaCl; 0.05% P20; 350 mM EDTA (30 s at 30 µl.min^−1^) was used to remove non-covalently attached protein, followed by quenching of the unreacted succinimide esters by an injection of 20 µl of 1 M H_2_N (CH_2_)_2_OH, pH 8.5 at 5 µl.min^−1^. Non-covalently bound proteins were washed off the surface with excess 10 mM HEPES, pH 7.5; 150 mM NaCl; 0.05% P20; 50 µM EDTA; 20 mM MgCl_2_ at 100 µl.min^−1^. Final protein immobilized levels were between ∼70 and 290 RU.

### SPR single cycle kinetic experiments

SPR single cycle kinetic titration binding experiments were performed at 25°C, using a 2-fold dilution series, in 10 mM HEPES, pH 7.5; 150 mM NaCl; 0.05% P20; 50 µM EDTA; 20 mM MgCl_2_ (or with buffer supplemented with 2 mM citrate) at 100 µl min^−1^ with a 60 seconds contact time and a 60 seconds dissociation time. The sensor surface was regenerated between experiments by dissociating any formed complex 10 mM HEPES, pH 7.5; 150 mM NaCl; 0.05% P20; 50 µM EDTA; 20 mM MgCl_2_ (or with buffer supplemented with 2 mM citrate). The apparent equilibrium dissociation constants (*K*_d_) were calculated from double reference corrected sensorgrams by global fitting of a 1∶1 binding model, including a mass transport term, using analysis software (v.1.0, GE Healthcare) provided with the BIAcore T200 instrument.

### Western blotting, flow cytometry and immunofluorescence

Protein expression analyses by western blotting and flow cytometry were carried out according to [21]. Immunofluorescence was carried out according to [13]. For quantitative scoring of differentiation in tsetse midguts the following criteria were used: ‘Bright’: homogenous, bright EP signal detected on the whole cell, ‘Faint’: faint and/or inhomogeneous EP signal detected on the whole cell (with a brighter flagellum and/or uneven, punctuated pattern); ‘Negative’- no EP signal detected. In all cases illumination and imaging settings were identical.

Phase–contrast and Immunofluorescence microscopy images were captured on a Zeiss axioskop2 (Carl Zeiss microimaging) with a Prior Lumen 200 light source using a QImaging Retiga 2000RCCD camera; the objective was a Plan Neofluar ×63 (1.25 NA). Images were captured *via* QImage (QImaging).

### Tsetse fly feeding and dissection

All tsetse flies were taken from the G*lossina morsitans morsitans* (Westwood) colony at LSTM, which is maintained at 26°C and 65–80% relative humidity, and fed on the supernatant of defibrinated horse blood. Male flies were fed only once, 24–72 hours after eclosion from the pupa. A minimum of 50 males were offered a feed on one of the meals described above for 10 minutes and were subsequently kept at 26–27°C for 4 hours. Flies were then chilled to 4°C and unfed flies removed. Flies with a meal in the gut were subsequently kept on ice until dissection.

For the EP expression experiment, the entire midgut from the proventriculus at the anterior end to the Malpighian tubules at the boundary with the hindgut was isolated in vPBS (8 g/l NaCl; 0.22 g/l KCl; 2.27 g/l Na_2_HPO_4_; 0.41 g/l KH_2_PO_4_; 15.7 g/l sucrose; 1.8 g/l glucose; pH 7.4). Dissected guts were then kept on ice in vPBS until all had been dissected. The guts were then gently disrupted using a micropestle in a 1.5 ml microcentrifuge tube. The content of the tube was then filtered through a 35 mm nylon mesh (BD Biosciences, England, #352235) to remove large pieces of midgut. The filtrate, which included trypanosomes, fly gut cells and fly gut bacteria was then washed twice in vPBS by pelleting at 836 *g* for 8 minutes and removing the supernatant. The cell pellet was then resuspended in around 600 µl vPBS and around 100 µl aliquots of the cell suspension was smeared over the surface of a polylysine treated slides and air dried.

### Preparation of the meals containing Soybean trypsin inhibitor

Horse serum was prepared by filtering through a 0.2 µm syringe filter the supernatant of defibrinated horse blood (TCS Biosciences Ltd., Buckingham, UK). Soybean (*Glycine max*) trypsin inhibitor (STI, Sigma Aldrich) was added to some aliquots of the serum at 1 mg/ml. All the serum aliquots were re-filtered and warmed to 37°C. Stumpy enriched populations of AnTat1.1 90:13 cells were mixed with 37°C HMI9 media and incubated for one hour at 37°C at 5% CO_2_. Cells were pelleted for 8 minutes at 836 *g* and resuspended in either 2 ml warmed horse serum or 2 ml warmed horse serum containing 1 mg/ml STI. The cells were then used immediately to feed tsetse flies after briefly checking cell viability by microscopy. The experiment was repeated three times, each time using a different batch of horse blood to prepare the serum. For the protease activity assays with *T. b. brucei* strain TSW196, aliquots of TSW196 blood from infected rats and mixed with 2 ml horse serum in the presence or absence of 1 mg/ml STI and immediately fed to tsetse flies.

Protease activity of midgut extracts was assayed using Chromozym TRY (Z-Val-Gly-Arg-pNA, Bachem, Switzerland) largely as described in [47].

## Supporting Information

Table S1 Oligonucleotides used in this study to generate mutant forms of *Tb*PIP39.
(DOCX)

Text S1 Supporting supplementary figures and legends referred to in this manuscript.
(PDF)

## References

[1] Rue P, Domedel-Puig N, Garcia-Ojalvo J, Pons AJ (2012) Integration of cellular signals in chattering environments. Prog Biophys Mol Biol 110 1: 106–12. 22584015 10.1016/j.pbiomolbio.2012.05.003

[2] Baena-Lopez LA, Nojima H, Vincent JP (2012) Integration of morphogen signalling within the growth regulatory network. Current opinion in cell biology 24: 166–172. 22257639 10.1016/j.ceb.2011.12.010

[3] Gallego-Bartolome J, Minguet EG, Grau-Enguix F, Abbas M, Locascio A, et al (2012) Molecular mechanism for the interaction between gibberellin and brassinosteroid signaling pathways in Arabidopsis. Proceedings of the National Academy of Sciences of the United States of America 109: 13446–13451. 22847438 10.1073/pnas.1119992109 PMC3421204

[4] Matthews KR (2011) Controlling and coordinating development in vector-transmitted parasites. Science 331: 1149–1153. 21385707 10.1126/science.1198077 PMC3834321

[5] Billker O, Shaw MK, Margos G, Sinden RE (1997) The roles of temperature, pH and mosquito factors as triggers of male and female gametogenesis of Plasmodium berghei in vitro. Parasitology 115 Pt 1: 1–7. 9280891 10.1017/s0031182097008895

[6] Sogin ML, Elwood HJ, Gunderson JH (1986) Evolutionary diversity of eukaryotic small-subunit rRNA genes. Proceedings of the National Academy of Sciences of the United States of America 83: 1383–1387. 2419907 10.1073/pnas.83.5.1383 PMC323080

[7] Brun R, Blum J, Chappuis F, Burri C (2010) Human African trypanosomiasis. Lancet 375: 148–159. 19833383 10.1016/S0140-6736(09)60829-1

[8] Zilberstein D, Shapira M (1994) The Role Of Ph and Temperature In the Development Of Leishmania Parasites. Annual review of microbiology 48: 449–470. 10.1146/annurev.mi.48.100194.002313 7826014

[9] Mittra B, Cortez M, Haydock A, Ramasamy G, Myler PJ, et al (2013) Iron uptake controls the generation of Leishmania infective forms through regulation of ROS levels. The Journal of experimental medicine 210: 401–416. 23382545 10.1084/jem.20121368 PMC3570109

[10] Ziegelbauer K, Quinten M, Schwarz H, Pearson TW, Overath P (1990) Synchronous differentiation of Trypanosoma brucei from bloodstream to procyclic forms in vitro. European journal of biochemistry/FEBS 192: 373–378. 10.1111/j.1432-1033.1990.tb19237.x 1698624

[11] Vickerman K (1985) Developmental cycles and biology of pathogenic trypanosomes. British medical bulletin 41: 105–114. 3928017 10.1093/oxfordjournals.bmb.a072036

[12] Bringaud F, Riviere L, Coustou V (2006) Energy metabolism of trypanosomatids: adaptation to available carbon sources. Mol Biochem Parasitol 149: 1–9. 16682088 10.1016/j.molbiopara.2006.03.017

[13] Dean SD, Marchetti R, Kirk K, Matthews K (2009) A surface transporter family conveys the trypanosome differentiation signal. Nature 459: 213–217. 19444208 10.1038/nature07997 PMC2685892

[14] Engstler M, Boshart M (2004) Cold shock and regulation of surface protein trafficking convey sensitization to inducers of stage differentiation in Trypanosoma brucei. Genes & development 18: 2798–2811. 15545633 10.1101/gad.323404 PMC528899

[15] MacGregor P, Matthews KR (2010) New discoveries in the transmission biology of sleeping sickness parasites: applying the basics. Journal of molecular medicine (Berlin, Germany) 88: 865–871. 10.1007/s00109-010-0637-y PMC2921060 20526573

[16] Laxman S, Riechers A, Sadilek M, Schwede F, Beavo JA (2006) Hydrolysis products of cAMP analogs cause transformation of Trypanosoma brucei from slender to stumpy-like forms. Proceedings of the National Academy of Sciences of the United States of America 103: 19194–19199. 17142316 10.1073/pnas.0608971103 PMC1748198

[17] Vassella E, Reuner B, Yutzy B, Boshart M (1997) Differentiation of African trypanosomes is controlled by a density sensing mechanism which signals cell cycle arrest via the cAMP pathway. Journal of cell science 110 Pt 21: 2661–2671. 9427384 10.1242/jcs.110.21.2661

[18] MacGregor P, Matthews KR (2012) Identification of the regulatory elements controlling the transmission stage-specific gene expression of PAD1 in Trypanosoma brucei. Nucleic Acids Res 40 16: 7705–17. 22684509 10.1093/nar/gks533 PMC3439917

[19] Barquilla A, Saldivia M, Diaz R, Bart JM, Vidal I, et al (2012) Third target of rapamycin complex negatively regulates development of quiescence in Trypanosoma brucei. Proceedings of the National Academy of Sciences of the United States of America 109: 14399–14404. 22908264 10.1073/pnas.1210465109 PMC3437835

[20] Szoor B, Wilson J, McElhinney H, Tabernero L, Matthews KR (2006) Protein tyrosine phosphatase TbPTP1: a molecular switch controlling life cycle differentiation in trypanosomes. The Journal of cell biology 175: 293–303. 17043136 10.1083/jcb.200605090 PMC2064570

[21] Szoor B, Ruberto I, Burchmore R, Matthews K (2010) A novel phosphatase cascade reglates differentiation in trypanosomes via a glycosomal signaling pathway. Genes and Development 24: 1306–1316. 20551176 10.1101/gad.570310 PMC2885665

[22] MacGregor P, Szoor B, Savill NJ, Matthews KR (2012) Trypanosomal immune evasion, chronicity and transmission: an elegant balancing act. Nature reviews 10: 431–438. 10.1038/nrmicro2779 PMC3834543 22543519

[23] Michels PA, Bringaud F, Herman M, Hannaert V (2006) Metabolic functions of glycosomes in trypanosomatids. Biochim Biophys Acta 1763 12: 1463–77. 17023066 10.1016/j.bbamcr.2006.08.019

[24] Rolin S, HanocqQuertier J, PaturiauxHanocq F, Nolan DP, Pays E (1998) Mild acid stress as a differentiation trigger in Trypanosoma brucei. Molecular and biochemical parasitology 93: 251–262. 9662709 10.1016/s0166-6851(98)00046-2

[25] Hunt M, Brun R, Kohler P (1994) Studies on compounds promoting the in vitro transformation of Trypanosoma brucei from bloodstream to procyclic forms. Parasitol Res 80: 600–606. 7855126 10.1007/BF00933009

[26] Sbicego S, Vassella E, Kurath U, Blum B, I R (1999) The use of transgenic Trypanosoma brucei to identify compounds inducing the differentiation of bloodstream forms to procyclic forms. Mol Biochem Parasitol 104: 311–322. 10593184 10.1016/s0166-6851(99)00157-7

[27] Yabu Y, Takayanagi T (1988) Trypsin-stimulated transformation of Trypanosoma brucei gambiense bloodstream forms to procyclic forms in vitro. Parasitol Res 74: 501–506. 3194362 10.1007/BF00531625

[28] Milne KG, Prescott AR, Ferguson MA (1998) Transformation of monomorphic Trypanosoma brucei bloodstream form trypomastigotes into procyclic forms at 37 degrees C by removing glucose from the culture medium. Mol Biochem Parasitol 94: 99–112. 9719513 10.1016/s0166-6851(98)00055-3

[29] Haanstra JR, Kerkhoven EJ, van Tuijl A, Blits M, Wurst M, et al (2011) A domino effect in drug action: from metabolic assault towards parasite differentiation. Mol Microbiol 79: 94–108. 21166896 10.1111/j.1365-2958.2010.07435.x

[30] Rolin S, Hanocq Q-J, Paturiaux H-F, Nolan D, Salmon D, et al (1996) Simultaneous but independent activation of adenylate cyclase and glycosylphosphatidylinositol-phospholipase C under stress conditions in Trypanosoma brucei. Journal of Biological Chemistry 271: 10844–10852. 8631899 10.1074/jbc.271.18.10844

[31] Nolan DP, Rolin S, Rodriguez JR, Van Den Abbeele J, Pays E (2000) Slender and stumpy bloodstream forms of Trypanosoma brucei display a differential response to extracellular acidic and proteolytic stress. European journal of biochemistry/FEBS 267: 18–27. 10.1046/j.1432-1327.2000.00935.x 10601846

[32] Creek DJ, Nijagal B, Kim DH, Rojas F, Matthews KR, et al (2013) Metabolomics Guides Rational Development of a Simplified Cell Culture Medium for Drug Screening against Trypanosoma brucei. Antimicrobial agents and chemotherapy 57: 2768–2779. 23571546 10.1128/AAC.00044-13 PMC3716122

[33] Yan J, Cheng Q, Li CB, Aksoy S (2001) Molecular characterization of two serine proteases expressed in gut tissue of the African trypanosome vector, Glossina morsitans morsitans. Insect molecular biology 10: 47–56. 11240636 10.1046/j.1365-2583.2001.00232.x

[34] Imbuga MO, Osir EO, Labongo VL, Darji N, Otieno LH (1992) Studies on tsetse midgut factors that induce differentiation of blood-stream Trypanosoma brucei brucei in vitro. Parasitol Res 78: 10–15. 1584740 10.1007/BF00936174

[35] Abubakar LU, Bulimo WD, Mulaa FJ, Osir EO (2006) Molecular characterization of a tsetse fly midgut proteolytic lectin that mediates differentiation of African trypanosomes. Insect biochemistry and molecular biology 36: 344–352. 16551548 10.1016/j.ibmb.2006.01.010

[36] Kamenski T, Heilmeier S, Meinhart A, Cramer P (2004) Structure and mechanism of RNA polymerase II CTD phosphatases. Molecular cell 15: 399–407. 15304220 10.1016/j.molcel.2004.06.035

[37] Brun R, Schonenberger M (1981) Stimulating effect of citrate and cis-Aconitate on the transformation of Trypanosoma brucei bloodstream forms to procyclic forms in vitro. Zeitschrift fur Parasitenkunde (Berlin, Germany) 66: 17–24. 10.1007/BF00941941 7324539

[38] Coughlin SR (2000) Thrombin signalling and protease-activated receptors. Nature 407: 258–264. 11001069 10.1038/35025229

[39] Berriman M, Ghedin E, Hertz-Fowler C, Blandin G, Renauld H, et al (2005) The genome of the African trypanosome Trypanosoma brucei. Science 309: 416–422. 16020726 10.1126/science.1112642

[40] McLintock LM, Turner CM, Vickerman K (1993) Comparison of the effects of immune killing mechanisms on Trypanosoma brucei parasites of slender and stumpy morphology. Parasite immunology 15: 475–480. 8233562 10.1111/j.1365-3024.1993.tb00633.x

[41] Chavel CA, Dionne HM, Birkaya B, Joshi J, Cullen PJ (2010) Multiple signals converge on a differentiation MAPK pathway. PLoS Genet 6: e1000883. 20333241 10.1371/journal.pgen.1000883 PMC2841618

[42] Fre S, Pallavi SK, Huyghe M, Lae M, Janssen KP, et al (2009) Notch and Wnt signals cooperatively control cell proliferation and tumorigenesis in the intestine. Proceedings of the National Academy of Sciences of the United States of America 106: 6309–6314. 19251639 10.1073/pnas.0900427106 PMC2649205

[43] Fu W, Baker NE (2003) Deciphering synergistic and redundant roles of Hedgehog, Decapentaplegic and Delta that drive the wave of differentiation in Drosophila eye development. Development 130: 5229–5239. 12954721 10.1242/dev.00764

[44] Kopf J, Petersen A, Duda GN, Knaus P (2012) BMP2 and mechanical loading cooperatively regulate immediate early signalling events in the BMP pathway. BMC biology [electronic resource] 10: 37. 10.1186/1741-7007-10-37 PMC3361481 22540193

[45] Hirumi H, Hirumi K (1989) Continuous cultivation of Trypanosoma brucei blood stream forms in a medium containing a low concentration of serum protein without feeder cell layers. The Journal of parasitology 75: 985–989. 2614608

[46] Brun R, Schönenburger M (1979) Cultivation and in vitro cloning of procyclyc forms of *Trypanosoma brucei* in a semi-defined medium. Acta tropica 36: 289–292. 43092

[47] Liniger M, Acosta-Serrano A, Van Den Abbeele J, Kunz Renggli C, Brun R, et al (2003) Cleavage of trypanosome surface glycoproteins by alkaline trypsin-like enzyme(s) in the midgut of Glossina morsitans. Int J Parasitol 33: 1319–1328. 14527515 10.1016/s0020-7519(03)00182-6

