# Supplementary material for: Independent Pathways Can Transduce the Life-Cycle Differentiation Signal in Trypanosoma brucei
Source: PLoS Pathog. 2013 Oct 17;9(10):e1003689. doi: 10.1371/journal.ppat.1003689 (PMC3798605; doi:10.1371/journal.ppat.1003689)
Supplement: Table S1 — Oligonucleotides used in this study to generate mutant forms of TbPIP39. (DOCX) [file ppat.1003689.s001.docx]

**Supplementary Table 1.** Oligonucleotides used in this study to generate mutant forms of *Tb*PIP39

| Oligonucleotid name | Sequence |
| --- | --- |
| Primer1  PIP39 D57A | 5'-acttgtgctggacatcgccgagacacttattcaca-3' |
| Primer2  PIP39 D57A antisense | 5'-tgtgaataagtgtctcggcgatgtccagcacaagt-3' |
| Primer3  PIP39 t63A v64A | 5’- acgagacacttattcacgcagcgggcatgcgcaacg -3' |
| Primer4  PIP39 t63A v64A antisense | 5’- cgttgcgcatgcccgctgcgtgaataagtgtctcgt -3' |
| Primer5  PIP39synthBamHI Fwd pGEX | 5’-cgttgcgcatgcccgctgcgtgaataagtgtctcgt -3' |
| Primer6  PIP39synthXhoI Rev pGEX | 5’-atactcgagctaaagacgacttggtgtgtgac -3' |
