# Supplementary material for: Independent Pathways Can Transduce the Life-Cycle Differentiation Signal in Trypanosoma brucei
Source: PLoS Pathog. 2013 Oct 17;9(10):e1003689. doi: 10.1371/journal.ppat.1003689 (PMC3798605; doi:10.1371/journal.ppat.1003689)
Supplement: Text S1 — Supporting supplementary figures and legends referred to in this manuscript. (PDF) [file ppat.1003689.s002.pdf]

Independent signalling mechanisms can transduce the life-cycle differentiation signal in *Trypanosoma brucei*

Balazs Szöőr, Naomi A. Dyer, Irene Ruberto, Alvaro Acosta Serrano and Keith R. Matthews

Supplementary Data

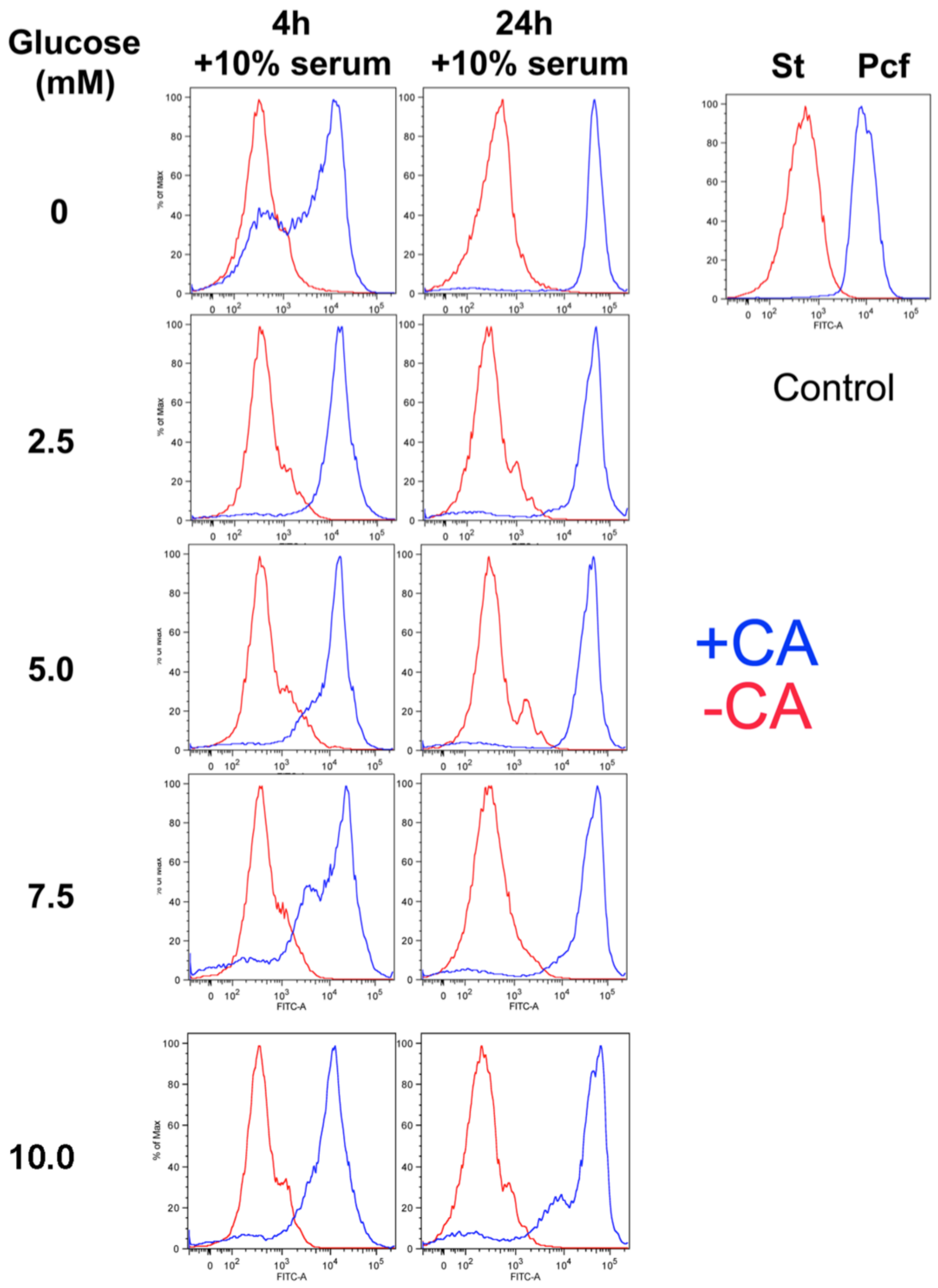

Figure S1

Glucose depletion does not stimulate the differentiation of stumpy form trypanosomes. Stumpy forms were incubated with 10% serum (approximately 0.5mM glucose final concentration) plus 0mM, 2.5mM, 5.0mM, 7.5mM or 10mM glucose in the presence (+CA) or absence (-CA) of 6mM *cis*-aconitate. In low glucose, no differentiation was observed, though the cells underwent efficient differentiation in the presence of CA. Without serum, the cells rapidly underwent cell death (not shown). Procyclin expression was monitored after 4h and 24h. Control assays were carried out also with stumpy form cells (red, right hand panel; negative control) or procyclic form cells (blue, right hand panel, positive control).

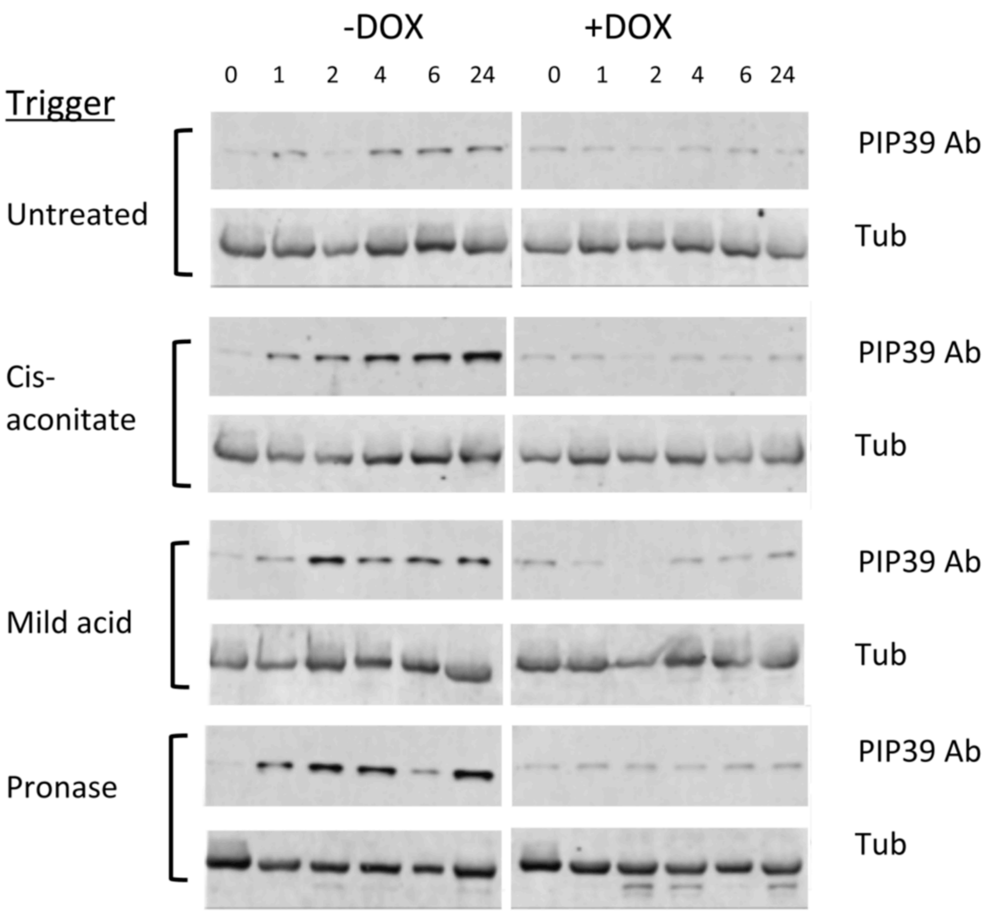

Figure S2

Western blots detecting *Tb*PIP39 from samples derived from *Tb*PIP39-RNAi stumpy cells, induced or uninduced with doxycycline during *in vivo* growth, and then exposed to cis-aconitate, mild acid, and pronase. Induction was retained after differentiation was stimulated by inclusion of tetracycline in the cell culture medium, with samples being prepared at the indicated times (in hours) thereafter. Western blots were reacted with the antibody against α-tubulin (Tub) to show the relative loading in each case, and with antibody detecting *Tb*PIP39.

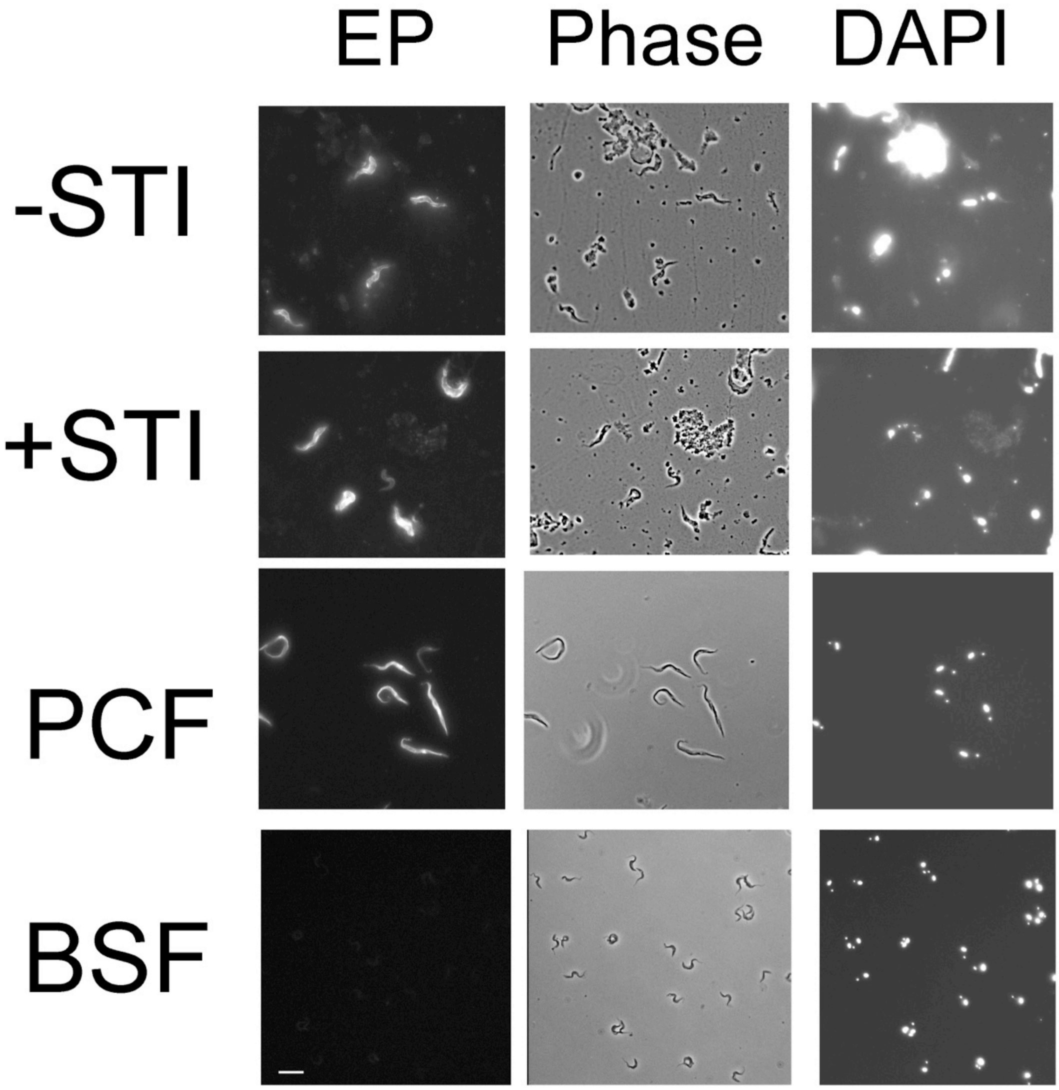

Figure S3

Trypanosomes in tsetse midgut extracts treated (+STI) or untreated (-STI) with Soybean trypsin inhibitor and probed for EP procyclin expression after 4 h. Controls of cultured procyclic forms and bloodstream forms are also shown. Exposures in the EP procyclin panels in each case were identical to allow comparison of relative staining. Bar= 10 µm.

```
PIP39     ATGGTGAGGACGACACGCTTTTCACGCAACAGTTCCAAACCACTGTTTCAGCAATTACAG 60
PIP39s    atggttcgcacaacacgctttagccgcaacagcagcaagccacttttcagcagctgcag
          M  V  R  T  T  R  F  S  R  N  S  S  K  P  L  F  Q  Q  L  Q   20

PIP39     CTTATTACATCGTATGGACTCTTTCAGGAGTATCCACCCATTTGGTCACTTCCCCTTCCA 120
PIP39s    ctgattacgagctacggtctttttcaagagtacccgccaatttggagccttccacttcca
          L  I  T  S  Y  G  L  F  Q  E  Y  P  P  I  W  S  L  P  L  P    40

PIP39     CCTCAACAACCTCGTGATACGGGAAAACCCACACTGGTGCTTGATATTGATGAAACACTG 180
PIP39s    ccacagcagccacgcgatacaggcaagccaacacttgtgctggacatcgacgagacactt
          P  Q  Q  P  R  D  T  G  K  P  T  L  V  L  D  I  D  E  T  L    60

PIP39     ATTCATACTGTGGGGATGCGGAATGAGGGGTCGGATTCTGTCTCCTTCAGTTTCTTCCTG 240
PIP39s    attcacacagtgggcatgcgcaacgagggtagtgatagtgtgtcgtttagctttttctg
          I  H  T  V  G  M  R  N  E  G  S  D  S  V  S  F  S  F  F  L    80

PIP39     CGTCCACATGCGAAGGAATTTCTCGCGGAGGTACGTGAACTGTACGAAGTTGTGTTCTGG 300
PIP39s    cgcccgcacgcgaaagaatttcttgcagaagtgcgcgagctgtacgaggtggtgttttgg
          R  P  H  A  K  E  F  L  A  E  V  R  E  L  Y  E  V  V  F  W   100

PIP39     ACGGCTGGTACCGCATCCTACTGCTCAGCTGTTATGGATGCACTGGAGGTGCAGGTGTTA 360
PIP39s    acagcaggcacagcaagttactgcagtgcagtgatggacgcgcttgaggtgcaagtgctt
          T  A  G  T  A  S  Y  C  S  A  V  M  D  A  L  E  V  Q  V  L   120

PIP39     CAACTCCCACGATCATTTTACAATATTGATGAACTCCGCGTAGAGGCACGTGGTGGAATC 420
PIP39s    cagcttccacgcagcttctacaacattgatgagcttcgtgtggaagcgcgcggtggtatt
          Q  L  P  R  S  F  Y  N  I  D  E  L  R  V  E  A  R  G  G  I   140

PIP39     TCCACCAAGAATGTTAACTTCTATGCCCTTTCCCGAACTCAAACGTTACAAGGGCATAAT 480
PIP39s    agtacgaagaacgtgaacttttacgcgcttagccgcacacagacacttcagggtcacaac
          S  T  K  N  V  N  F  Y  A  L  S  R  T  Q  T  L  Q  G  H  N   160

PIP39     TACATGAAATACCTTCCAATGCTTGGCAGACCATTGAATCGTGTCATAGTGATCGACAGT 540
PIP39s    tacatgaagtacttgccgatgcttggtcgcccgcttaaccgtgtgattgtgattgatagc
          Y  M  K  Y  L  P  M  L  G  R  P  L  N  R  V  I  V  I  D  S   180

PIP39     GATGTACGCAGCTTTCCTCTTCATCCACGTAACGGTGTTAAGATCGAACCCTTCCTACCG 600
PIP39s    gacgtgcgcagttttccattgcacccacgtaatggcgtgaagattgagccatttcttcca
          D  V  R  S  F  P  L  H  P  R  N  G  V  K  I  E  P  F  L  P   200

PIP39     AATGAACGTGTTTTATCCGAATATGCCCGCGTAGTAACCGATGAGGTGAAATATGGTCAA 660
PIP39s    aacgagcgcgtgttgagcgagtacgcacgtgtggttacggatgaagtgaagtacggtcaa
          N  E  R  V  L  S  E  Y  A  R  V  V  T  D  E  V  K  Y  G  Q   220

PIP39     GTTGGTCAGCGGCAGTATGAAGGCGAAATGGCAAAAGTCATTGAGCGTGGTGAGGAGGAG 720
PIP39s    gtgggacaacgccagtacgagggggagatggcgaaagtgattgaacgcggagaggaagag
          V  G  Q  R  Q  Y  E  G  E  M  A  K  V  I  E  R  G  E  E  E   240

PIP39     GTAGCGCGATTGCAAGCGGATCATGCCCTTATGGATCTGATTCCAATGCTACGCTCTGCG 780
PIP39s    gtggcacgccttcaggcagatcacgcattgatggaccttattccgatgcttcgcagtgca
          V  A  R  L  Q  A  D  H  A  L  M  D  L  I  P  M  L  R  S  A   260

PIP39     GCTTCTTCAACTGATTTGACTCACGAATTAGACCATTGGCGTACGGATGAGTACACAAAG 840
PIP39s    gcaagcagcacagatcttacacacgagcttgatcactggcgcacagacgagtacacgaaa
          A  S  S  T  D  L  T  H  E  L  D  H  W  R  T  D  E  Y  T  K   280

PIP39     TGTGACGACTTTCGTGAAACGATGAACTCTCTCTCAGTGACAAGGCAGAAGATTCTTGGT 900
PIP39s    tgtgatgatttccgcgagacaatgaacagccttagtgtgacgcgccaaaagattctgggc
          C  D  D  F  R  E  T  M  N  S  L  S  V  T  R  Q  K  I  L  G   300

PIP39     AATGTTCTTAAGGAGCGACGCAACGTACCGATTCCGCCTCTCAAGCAGCACGTGATGAAT 960
PIP39s    aacgtgctgaaagaacgccgtaacgtgccaattccaccgttgaagcaacatgtgatgaac
          N  V  L  K  E  R  R  N  V  P  I  P  P  L  K  Q  H  V  M  N   320

PIP39     CATGGATTTATGGAGGAGGCAAATGCCGCTATGAAGTTGGAGCAGATGCGACACACTCCT 1020
PIP39s    cacggtttcatggaagaggcgaacgcagcaatgaagctcgagcaaatgcgtcacacacca
          H  G  F  M  E  E  A  N  A  A  M  K  L  E  Q  M  R  H  T  P    340

PIP39     TCAAGACTTTAG 1032
PIP39s    agtcgtctt
          S  R  L      343
```

Figure S4

Gene sequence of *Tb*PIP39 and recoded *Tb*PIP39 mutants where predicted citrate binding residues (in bold and underlined) are mutated

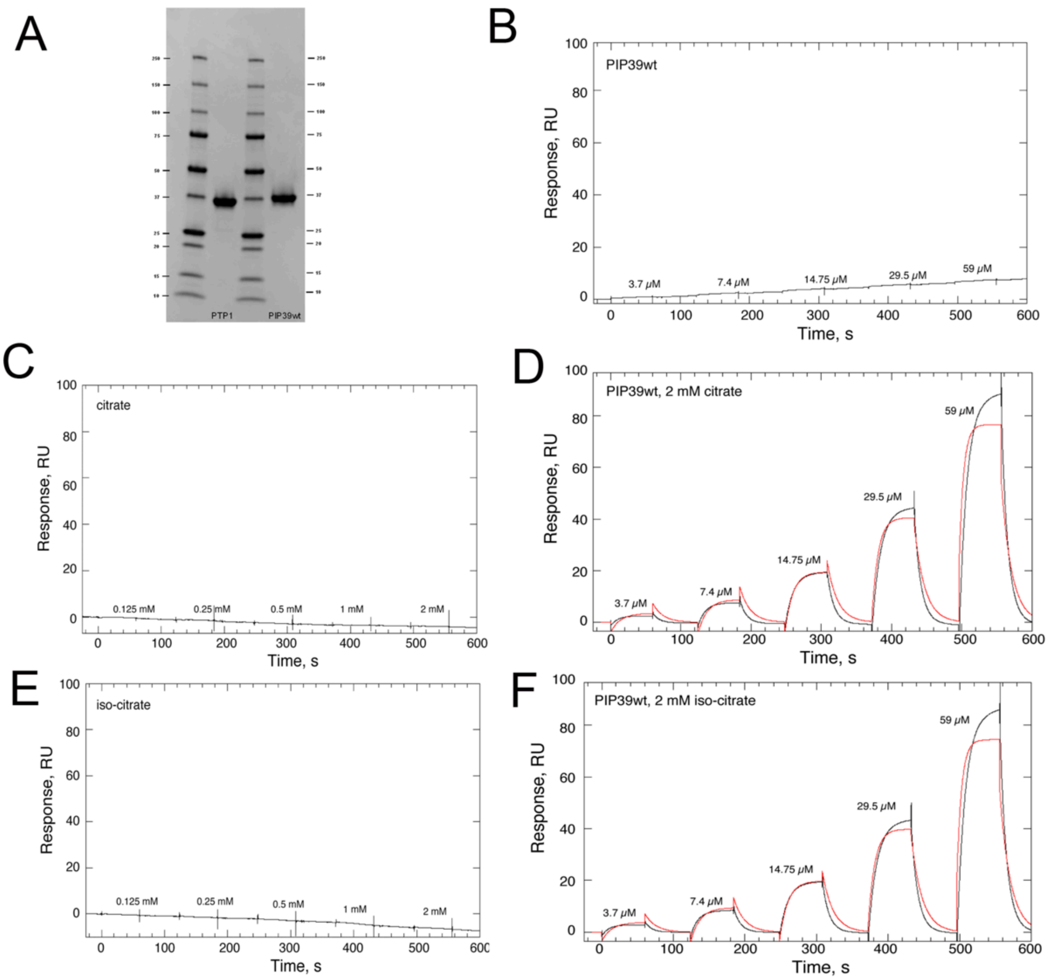

Figure S5

Citrate and isocitrate allow interaction between *Tb*PIP39 and *Tb*PTP1.

A. Purified *Tb*PTP1 and *Tb*PIP39wt used in these interaction assays
B. 2-fold dilution of PIP39wt from 59 μM - 3.7 μM in the absence of either citrate or iso-citrate subjected to surface plasmon resonance on a *Tb*PTP1 surface and analysed using a T200 BIAcore.
C. 2-fold dilution of citrate alone from 2 mM - 0.125 mM subjected to surface plasmon resonance on a *Tb*PTP1 surface and analysed using a T200 BIAcore.
D. 2-fold dilution of *Tb*PIP39wt from 59 μM - 3.7 μM in the presence of 2 mM citrate subjected to surface plasmon resonance on a *Tb*PTP1 surface and analysed using a T200 BIAcore.
E. 2-fold dilution of iso-citrate alone from 2 mM - 0.125 mM subjected to surface plasmon resonance on a *Tb*PTP1 surface and analysed using a T200 BIAcore.
F. 2-fold dilution of PIP39wt in from 59 μM - 3.7 μM in the presence of 2 mM iso-citrate subjected to surface plasmon resonance on a *Tb*PTP1 surface and analysed using a T200 BIAcore.

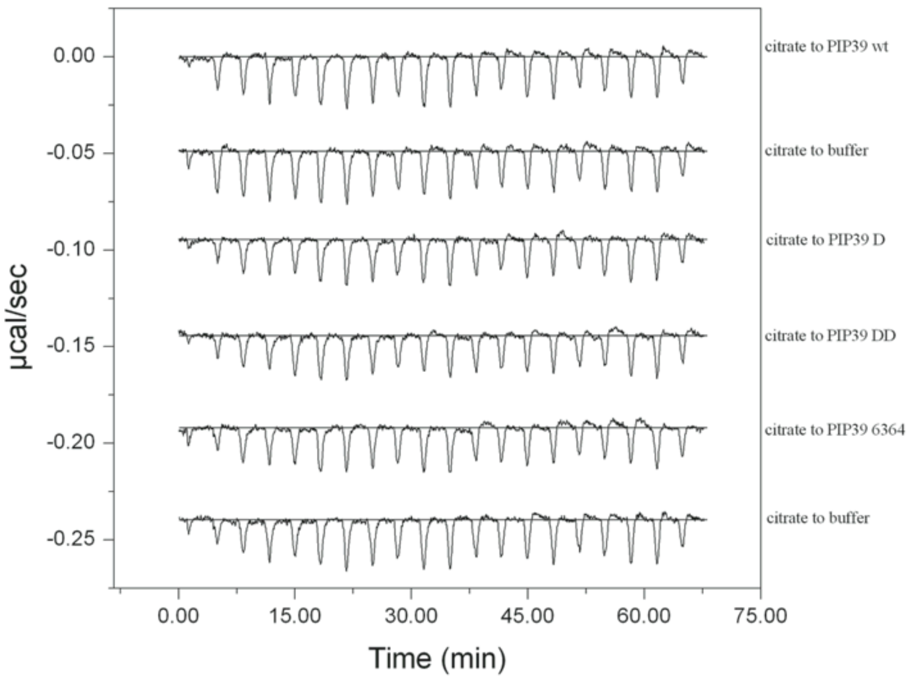

Figure S6

Calorimetric titration of citrate into recombinant TbPIP39 wt, D, DD and 6364 mutants.

*Tb*PIP39 wt, D, DD or 6364 mutants were desalted into *ITC buffer* (10 mM Hepes, pH7.3; 0.05% P20; 20 mM $MgCl_2$ and 2mM DTT) on a pre-equilibrated 5ml HiTrap desalting column (GE Healthcare). After a preliminary injection of 2 µl, nineteen 15 µl aliquots of 2 mM citrate in *ITC buffer* were injected into 1.41 ml of 5 µM *Tb*PIP39 wt, D, DD or 6364 mutants at 25 °C using an AutoITC instrument (MicroCal). The experimental parameters were as follows: injection time 30s; 200s inter-injection delay, stirring 250 rpm and power setting 5. Control experiments were carried out in which 2 mM citrate in ITC buffer was injected into ITC buffer to determine heat of dilution for 2 mM citrate in ITC buffer. Each peak represents the injection of a 15 µl aliquot of 2 mM citrate into 1.41 ml of 5 µM *Tb*PIP39 at 25 °C in 10 mM Hepes, pH7.3; 0.05% P20; 20 mM $MgCl_2$ and 2mM DTT. No difference was observed in the presence of *Tb*PIP39 or buffer only, demonstrating an absence of detectable citrate binding.

For Figures S5 and S6 recombinant proteins were produced and purified as follows: Recombinant proteins were expressed and purified to homogeneity from BL21 (DE3) Star *E. coli* (Invitrogen), grown shaking (260 rpm) at 30 ˚C for 16 hrs in EnPresso Medium (BioSilta) containing carbenicillin (100 µg.ml^{-1}). Cell pellets of His-PTP1 were resuspended in 20 mM NaH_2PO_4, pH 7.4; 500 mM NaCl; 20 mM Imidazole; plus protease inhibitors at 10 % wt/vol, and lysed

by a single passage through a Constant Systems Cell Disruptor (1.1 kW TS Benchtop) set at 22 kpsi, followed by centrifugation at 50,000*g* for 1hr at 4 ˚C. The filtered (0.22 µm) supernatant was loaded onto an ÅKTA Purifier (GE Healthcare) fitted with a 1ml HiTrap IMAC FF column (GE Healthcare) and an HiPrep S200 16/60 HR (GE Healthcare), run with standard configuration and settings for a 2-step affinity-gel-filtration protocol. Buffers used for the purification were; *IMAC Loading buffer*: 20 mM NaH_2PO_4, pH 7.4; 500 mM NaCl; 20 mM Imidazole; 100 µM PMSF. *IMAC Elution buffer:* 20 mM NaH_2PO_4, pH 7.4; 500 mM NaCl; 500 mM Imidazole. *Gel-Filtration Buffer*: 10 mM HEPES, pH 7.5; 150 mM NaCl; 0.05% P20; 50 µM EDTA; 20 mM $MgCl_2$. GST-PIP39wt, GST-PIP39 D, GST-PIP39 DD and GST-PIP39 6364 were resuspended in 20 mM HEPES, pH7.5; 150 mM NaCl, 1mM DTT; 5 % glycerol plus protease inhibitors at 10 % wt/vol, and lysed by a single passage through a Constant Systems Cell Disruptor (1.1 kW TS Benchtop) set at 22 kpsi, followed by centrifugation at 50,000 x g for 1hr at 4 °C. The filtered (0.22 µm) supernatant was loaded onto an ÅKTA Purifier (GE Healthcare) fitted with a 1ml GSTrap FF column (GE Healthcare) and an HiPrep S200 16/60 HR (GE Healthcare), run with standard configuration and settings for a 2-step affinity-gel-filtration protocol. Buffers used for the purification were; *GSTrap Loading buffer*: 20 mM HEPES, pH7.5; 150 mM NaCl, 1mM DTT; 5 % glycerol. *GSTrap Elution buffer* 20 mM HEPES, pH7.5; 150 mM NaCl, 1mM DTT; 5 % glycerol; 20 mM reduced glutathione. *Gel-Filtration Buffer*: 10 mM HEPES, pH 7.5; 150 mM NaCl; 0.05% P20; 50 µM EDTA; 20 mM $MgCl_2$.
